# Metabolic reprogramming-driven stratification and therapeutic targeting in lung adenocarcinoma: implications for prognosis and personalized treatment

**DOI:** 10.3389/fonc.2025.1696117

**Published:** 2026-01-05

**Authors:** Jiabao Jia, Shiyun Feng, Rui Wang, Xiaoshuang Zhao, Youbin Cui

**Affiliations:** 1 Department of Thoracic Surgery, The First Hospital of Jilin University, Changchun, Jilin, China; 2 Department of Nursing, The First Hospital of Jilin University, Changchun, Jilin, China

**Keywords:** lung adenocarcinoma, metabolic reprogramming, prognostic signature, immune microenvironment, immunotherapy

## Abstract

**Background:**

Metabolic reprogramming is a hallmark of lung adenocarcinoma (LUAD) progression and therapy resistance, yet its clinical integration for prognosis and treatment remains limited. This study aimed to develop a metabolism-related prognostic score (MRPs) and investigate its biological, immunological, and therapeutic implications in LUAD.

**Methods:**

Transcriptomic and clinical data from TCGA and GEO were used to construct a prognostic model based on metabolism-associated genes *via* LASSO-Cox regression. The model was validated in internal and external cohorts. Functional enrichment, immune infiltration analysis, drug sensitivity prediction (TIDE), and single-cell RNA sequencing were performed. WARS2 was identified as a key gene and validated through qPCR, Western blot, immunohistochemistry, and functional assays *in vitro* and *in vivo*.

**Results:**

The MRPs model stratified LUAD patients into two subgroups with distinct prognoses. The high-risk MRP I subgroup showed enhanced oxidative phosphorylation, cell cycle activity, and protein synthesis, along with suppressed immune infiltration and lower immune scores. WARS2 was highly expressed in malignant epithelial cells and associated with proliferative and metabolic programs at both bulk and single-cell levels. WARS2-positive cells exhibited increased intercellular communication *via* adhesion-related pathways. Functional assays confirmed that WARS2 silencing impaired LUAD cell proliferation, invasion, and tumor growth.

**Conclusion:**

This study presents a novel MRPs system that captures LUAD metabolic and immune heterogeneity and surpasses traditional clinicopathologic parameters in prognostic performance. WARS2 emerges as a functional driver and therapeutic target. These findings offer a comprehensive framework for metabolic-state–based stratification and personalized treatment strategies in LUAD.

## 1 Introduction

Lung cancer is the foremost cause of cancer-related deaths globally (1), accounting for approximately 1.8 million mortality annually (2). Among its subtypes, lung adenocarcinoma (LUAD) constitutes the most prevalent histological form of non-small cell lung cancer (NSCLC) and poses a significant clinical challenge due to its aggressive biological behavior and marked heterogeneity (3, 4). Despite considerable advances in surgical techniques, radiochemotherapy, targeted molecular agents (such as EGFR and ALK inhibitors), and immune checkpoint inhibitors (ICIs) (5–9), the long-term prognosis for patients with advanced LUAD remains dismal (10). The five-year overall survival rate continues to hover below 20%, particularly in cases diagnosed at late stages, underscoring the limitations of current diagnostic and therapeutic strategies (11). A major contributor to this therapeutic impasse is the profound inter- and intra-tumoral heterogeneity observed in LUAD, which leads to diverse biological phenotypes, variable treatment responses, and the emergence of adaptive resistance (12). Consequently, the identification of robust, biologically informed molecular classifiers capable of stratifying LUAD patients and guiding personalized therapeutic interventions has become an urgent priority in precision oncology (13).

Among the hallmarks of cancer, metabolic reprogramming has emerged as a core feature that enables tumor cells to adapt to dynamic microenvironmental stresses, sustain bioenergetic and biosynthetic demands, and evade immune surveillance (14–16). Through the reshaping of key metabolic pathways, including aerobic glycolysis (the Warburg effect), glutaminolysis, fatty acid synthesis, and mitochondrial oxidative phosphorylation—cancer cells gain a selective growth advantage and develop resistance to various therapies (17–20). Importantly, metabolic rewiring not only supports intrinsic tumor proliferation and survival but also profoundly influences the tumor microenvironment (TME) by promoting immune evasion, suppressing antigen presentation, altering cytokine networks, and reshaping stromal interactions (21, 22).

In LUAD, emerging evidence underscores the dual role of metabolic remodeling: it simultaneously drives tumor aggressiveness and orchestrates an immunosuppressive milieu that diminishes responsiveness to immunotherapy and chemotherapy (23). For example, lactate accumulation, glutamine addiction, and altered redox balance have all been implicated in promoting immune dysfunction and facilitating tumor escape (24–26). These insights position metabolic reprogramming at a pivotal intersection of tumor evolution, immune regulation, and therapeutic resistance.

Nevertheless, most existing studies focus on isolated metabolic pathways or single gene events, lacking a systems-level perspective that integrates global metabolic signatures into clinically actionable frameworks (27, 28). The translational utility of metabolism-based classifiers in guiding prognosis and therapy selection—especially in the context of immune-oncology—remains largely underexplored in LUAD.

To address this critical gap, the present study leverages transcriptomic, clinical, and functional data to construct a robust Metabolic Reprogramming-associated Prognostic Signature (MRPs) for LUAD. Utilizing machine learning algorithms and systematic bioinformatics pipelines, we stratify patients based on metabolic expression profiles and evaluate their associations with survival outcomes, immune infiltration patterns, potential therapeutic sensitivities, and predicted responses to immunotherapy. Moreover, we identify the mitochondrial tryptophanyl-tRNA synthetase gene WARS2 as a pivotal metabolic regulator and validate its functional role using single-cell transcriptomics, *in vitro* experiments, and *in vivo* xenograft models.

Altogether, this study establishes a comprehensive metabolism-centered stratification system that not only enhances the prognostic resolution for LUAD patients but also illuminates novel mechanistic underpinnings and therapeutic vulnerabilities. By integrating metabolic phenotypes with immune contexture and drug response patterns, our findings provide a conceptual and practical framework for advancing next-generation precision oncology in LUAD.

## 2 Materials and methods

### 2.1 Data acquisition and preprocessing

This research utilized transcriptomic data of lung adenocarcinoma (LUAD) from The Cancer Genome Atlas (TCGA) and single-cell RNA sequencing data from the GEO dataset GSE148071. TCGA-LUAD expression data were downloaded from the UCSC Xena platform, comprising 530 LUAD tumor samples and 50 adjacent normal lung tissue samples. Both the HTSeq-count raw read counts and TPM (Transcripts Per Million) normalized expression matrices were obtained, along with corresponding clinical annotation files for downstream analysis. The GSE148071 dataset was retrieved from Gene Expression Omnibus (GEO, which were used to explore the expression patterns and functional roles of target genes across distinct cell types within the tumor microenvironment. To systematically investigate the role of metabolic reprogramming in LUAD, we extracted all metabolic pathways from the KEGG database that included the keyword “metabolism” in their pathway names. All genes involved in these pathways were compiled to construct a metabolism-related gene set, which served as the basis for subsequent functional annotation and expression analyses.

### 2.2 DEGs recognition

Differential expression genes recognition was performed using the DESeq2 package in R. Raw count data in HTSeq-count format were used as input, and sample group information (e.g., tumor vs. normal) was incorporated to construct a DESeqDataSet object. After normalization and dispersion estimation, a negative binomial generalized linear model was fitted to the data to identify DEGs. Multiple testing correction was performed using the Benjamini–Hochberg method to control the false discovery rate (FDR). Genes with an absolute log2 fold change > 1 and FDR < 0.05 were regarded as significantly differentially expressed. The identified DEGs were subjected to downstream analyses, including enrichment analysis, visualization, and functional interpretation.

### 2.3 Construction and evaluation of a prognostic signature based on MRPGs

To develop a prognostic model associated with metabolic reprogramming in LUAD, we utilized the previously curated metabolic reprogramming-related genes (MRPG), derived from KEGG pathways containing the keyword “metabolism.” A total of 530 TCGA-LUAD patients were randomly divided into a training set and a testing set at a ratio of 7:3. In the training cohort, univariate Cox proportional hazards regression was performed to identify MRPGs significantly associated with overall survival (OS) (P < 0.05). These candidate genes were then subjected to least absolute shrinkage and selection operator (LASSO) regression for dimensionality reduction and feature selection. Genes retained in the multivariate Cox regression model were used to construct a metabolic reprogramming score (MRPs). The MRPs for each sample were calculated following:


$$MRPs=\sum_{i=1}^{n}Coef_{i}\times Expr_{i}$$

Where Coefi represents the regression coefficient of gene i from the multivariate Cox model, and Expri denotes its expression level in each sample.

Patients were then stratified into two groups based on the median MRP score: high-risk (MRP I group) and low-risk (MRP II group). Kaplan–Meier survival analysis was applied to compare overall survival between MRPs I and MRPs II. Time-dependent receiver operating characteristic (ROC) curves were generated to examine the predictive performance of the model at 1-, 3-, and 5-year time points. The testing set was employed to independently validate the robustness and generalizability of the prognostic model.

### 2.4 Nomogram construction and validation

To facilitate individualized survival prediction, we constructed a nomogram integrating the metabolic reprogramming-based risk score and key clinical parameters, including age, sex, and tumor stage. The nomogram was generated using the “rms” package in R. This graphical prediction tool allows estimation of 1-, 3-, and 5-year overall survival probabilities by assigning a weighted score to each variable. The predictive accuracy and calibration of the nomogram were assessed through time-dependent calibration curves. These plots compared the predicted survival probabilities with actual observations, providing a visual evaluation of the model’s reliability and clinical utility.

### 2.5 Tumor mutation landscape analysis

To investigate the somatic mutation landscape in LUAD, we utilized the “maftools” package in R to analyze and visualize mutation annotation format (MAF) data. Waterfall plots were generated to display the most frequently mutated genes in the cohort, along with the genes included in our prognostic model. Patients were stratified into MRP I and MRP II groups according to their metabolic reprogramming scores. Tumor mutation burden (TMB) was calculated for each sample based on the total number of somatic mutations. Comparisons of TMB levels between the two groups were conducted to assess potential associations between mutation load and metabolic classification.

### 2.6 Functional enrichment analysis of MRPGs

To explore the biological significance of metabolic reprogramming-associated transcriptional alterations, we performed functional enrichment analysis on the differentially expressed genes (DEGs) identified between the MRP I and MRP II groups. Gene Ontology (GO) and Kyoto Encyclopedia of Genes and Genomes (KEGG) pathway analyses were conducted to annotate the DEGs in terms of biological processes, molecular functions, and signaling pathways. An adjusted false discovery rate (FDR) threshold of < 0.05 was applied to identify significantly enriched terms. Visualization of the enrichment results was carried out using the R packages “enrichplot” (version 1.20.3) and “ggplot2” (version 0.4.9).

### 2.7 Pathway activity and functional enrichment analysis *via* GSVA and GSEA

To investigate the underlying biological differences between MRP I and MRP II groups, we employed two complementary gene set–based approaches: Gene Set Variation Analysis (GSVA) and Gene Set Enrichment Analysis (GSEA). GSVA was performed to estimate the variation in pathway activity across individual patients in an unsupervised manner. It transformed the gene expression matrix into pathway-level enrichment scores, enabling a sample-wise comparison of metabolic and signaling pathway activities. In parallel, we applied GSEA to identify biological processes and signaling cascades that were significantly enriched between the two groups. Genes were ranked based on their differential expression statistics between MRP I and MRP II, and enrichment scores were calculated using curated gene sets. For GSVA, we used KEGG pathway gene sets to infer functional states, while GO biological process gene sets were adopted for GSEA. Additionally, to systematically assess the activity of canonical oncogenic signaling pathways, we applied the “progeny” R package, which uses predefined transcriptional footprints to infer pathway activation levels. This multi-layered analysis provided robust insights into distinct biological programs associated with metabolic reprogramming status in LUAD patients.

### 2.8 Evaluation of immune infiltration and the tumor immune microenvironment

To characterize the immune landscape of LUAD patients, we applied two computational approaches: the ESTIMATE algorithm and single-sample Gene Set Enrichment Analysis (ssGSEA). The ESTIMATE algorithm was used to infer the immune score, stromal score, tumor purity, and overall ESTIMATE score for each patient based on gene expression signatures. These scores provided a global assessment of the immune and stromal components within the tumor microenvironment. Additionally, ssGSEA was performed to quantify the enrichment levels of 29 predefined immune-related gene sets for each individual sample, capturing the activity of diverse immune functions and cell populations. This analysis enabled the profiling of immune states across samples. Differences in immune scores and enrichment profiles between MRP I and MRP II groups were then compared to evaluate potential associations between metabolic reprogramming status and immune infiltration patterns.

### 2.9 Drug sensitivity analysis

To explore potential therapeutic agents and assess differential drug responses, we applied the oncoPredict package (version 0.2) to estimate the half-maximal inhibitory concentration (IC50) values of 198 antitumor compounds across all samples. The predicted IC50 values provided a measure of each sample’s sensitivity to the corresponding drugs. Subsequently, we compared the IC50 distributions between the MRP I and MRP II groups to identify compounds with significantly different sensitivities. Drugs with a mean IC50 value below 1 across all samples were prioritized for further investigation, as they may represent more potent candidates for metabolic reprogramming–stratified treatment strategies.

### 2.10 Single-cell RNA-seq data processing and analysis

The single-cell RNA sequencing dataset GSE148071 was downloaded from the GEO database and preprocessed using the Seurat (v4.3.0) R package. Cells with fewer than 200 detected genes or with over 10% mitochondrial gene content were filtered out to ensure data quality. Genes expressed in fewer than three cells were also excluded. After normalization and scaling, highly variable genes were identified, and dimensionality reduction was performed using principal component analysis (PCA). Uniform Manifold Approximation and Projection (UMAP) was subsequently applied for visualization. Cell clustering was conducted using the Louvain algorithm, and clusters were annotated into distinct cell types based on canonical marker genes and reference-based mapping using the SingleR package. Following cell-type identification, differentially expressed genes (DEGs) between specific groups or cell states were determined using the Wilcoxon rank-sum test embedded in Seurat, with adjusted p-value < 0.05 and |log2FC| > 0.25 as thresholds. To evaluate pathway activity at the single-cell level, we employed the AUCell algorithm to score predefined gene sets within individual cells. Gene sets of interest, particularly those related to metabolism or immune function, were used to assess their activation across various cell types or states. For cell-cell communication analysis, we used the CellChat package (v1.6.1), which predicts intercellular signaling based on known ligand-receptor interactions. We constructed a CellChat object from the normalized expression matrix and applied the default pipeline to infer communication probability, identify major signaling pathways, and visualize interaction networks, focusing on pathways enriched in WARS2-positive cell populations. Single-cell RNA sequencing data of lung adenocarcinoma (LUAD) were obtained from the GSE148071 dataset (GEO). Among the 42 NSCLC samples, 18 LUAD samples were selected based on metadata annotations (‘histology: lung adenocarcinoma’). After quality control, approximately 38,000 high-quality cells were retained for downstream analyses.

### 2.11 Immunohistochemical validation using the Human Protein Atlas

To validate the protein-level expression of WARS2, we retrieved immunohistochemistry (IHC) images from the Human Protein Atlas (HPA, https://www.proteinatlas.org/). A total of three normal lung tissue samples and three lung adenocarcinoma (LUAD) tissue samples were selected for comparison. These images were used to visually assess the differential expression of WARS2 between normal and tumor tissues, providing additional confirmation of the transcriptomic patterns observed in our analysis.

### 2.12 LUAD cell lines culture

Human lung adenocarcinoma cell lines A549 and PC9 were used for *in vitro* experiments. Both cell lines were procured from Wuhan Procell Life Science & Technology Co., Ltd. (Wuhan, China). Cells were cultured in RPMI-1640 medium (Gibco, USA) supplemented with 10% fetal bovine serum (FBS) (Gibco, USA) and 1% penicillin-streptomycin under standard conditions (37°C, 5% CO_2_). The medium was refreshed every 2–3 days, and cells were passaged at approximately 80–90% confluency using 0.25% trypsin-EDTA.

### 2.13 Cell transfection

LUAD cell lines were seeded into 6-well plates and cultured until reaching approximately 70–80% confluency. Transfection was performed using Lipofectamine 2000 (Invitrogen, Cat. No. 11668019) in accordance with the manufacturer’s instructions. Briefly, siRNA and Lipofectamine 2000 were diluted in Opti-MEM I Reduced Serum Medium (Gibco, Cat. No. 31985070) and incubated at room temperature to allow complex formation. Prior to transfection, the culture medium was replaced with antibiotic-free RPMI-1640. The transfection complexes were then added to the cells and incubated under standard conditions. After 6 hours, the medium was replaced with complete RPMI-1640 supplemented with 10% fetal bovine serum and 1% penicillin–streptomycin. Cells were harvested for downstream assays at designated time points.

### 2.14 qPCR

Total RNA was extracted from LUAD cell lines using TRIzol reagent (Beyotime, Cat. No. R0016, Shanghai, China). The purity and concentration of RNA were assessed using a NanoDrop spectrophotometer (Thermo Scientific, Shanghai, China). Subsequently, cDNA synthesis was carried out using the High-Capacity cDNA Reverse Transcription Kit (Thermo, Cat. No. 4387406, Shanghai, China), with 1 μg of total RNA as input. Quantitative real-time PCR was performed using SYBR Green PCR Master Mix (Servicebio, Cat. No. G3322-01, Wuhan, China) on a CFX Connect Real-Time PCR Detection System (Bio-Rad, Shanghai, China). Gene expression levels were normalized to GAPDH as the internal control, and relative expression was calculated using the 2^−ΔΔCt method.

### 2.15 Western blot analysis

Total protein was extracted from LUAD cell lines using RIPA lysis buffer supplemented with protease and phosphatase inhibitors (Servicebio, Cat. No. G2002-100ML, Wuhan, China). Protein concentrations were quantified using a BCA Protein Assay Kit (Beyotime, Cat. No. P0012, Shanghai, China) following the manufacturer’s protocol. Equal amounts of protein were resolved by SDS–PAGE and subsequently transferred onto polyvinylidene fluoride (PVDF) membranes (Cytiva, Cat. No. 10600023, Shanghai, China). Membranes were blocked with 5% non-fat milk in TBST (Tris-buffered saline with 0.1% Tween-20) for 1 hour at room temperature to prevent nonspecific binding. The membranes were incubated overnight at 4 °C with primary antibodies against WARS2 (Proteintech, Cat. No. 15617-1-AP, Wuhan, China), COXII (CST, Cat. No. 4824S), ATP5A (Proteintech, Cat. No. 14676-1-AP), p-AMPK (Proteintech, Cat. 83924-1-RR) and GAPDH (Proteintech, Cat. No. 10494-1-AP, Wuhan, China) diluted in blocking buffer. After washing, membranes were incubated with HRP-conjugated secondary antibodies (Proteintech, Cat. No. SA00001-2, Wuhan, China) for 1 hour at room temperature. Protein signals were visualized using an enhanced chemiluminescence (ECL) detection system (Bio-Rad ChemiDoc MP, Shanghai, China). Band intensities were quantified using ImageJ software (version 1.54g, NIH, USA) to assess relative WARS2 protein expression levels.

### 2.16 Flow cytometry

Single-cell suspensions were prepared from freshly excised xenograft tumors using collagenase IV and DNase I digestion. After filtration through 70-μm strainers, cells were washed and stained with fluorochrome-conjugated antibodies against CD3 and CD8 (for cytotoxic T cells) or CD45 and CD206 (for M2-like macrophages) for 30 min at 4 °C in the dark. Samples were acquired on a BD LSRFortessa flow cytometer and analyzed using FlowJo v10 software. The proportions of CD3+ CD8+ T cells and CD45+ CD206+ macrophages were quantified, and results were expressed as the percentage of parent populations.

### 2.17 Cell proliferation assays

Cell proliferation was assessed using both the Cell Counting Kit-8 (CCK-8) assay and the 5-ethynyl-2′-deoxyuridine (EdU) incorporation assay. For the CCK-8 assay, LUAD cell lines were seeded into 96-well plates at a density of 5,000 cells per well and cultured under standard conditions. After treatment with the indicated conditions for the specified time points, the culture medium was replaced with fresh medium containing 10% CCK-8 reagent (Beyotime, Cat. No. C0037, Shanghai, China) and incubated at 37°C with 5% CO_2_ for 1 hour. Absorbance at 450 nm was measured using a microplate reader (Thermo, Cat. No. A51119600C, Shanghai, China), and the optical density values were used to determine relative cell viability. For the EdU assay, cells were seeded in 24-well plates and treated as indicated. DNA synthesis was detected using an EdU assay kit (Beyotime, Cat. No. C0071S, Shanghai, China) following the manufacturer’s instructions. Briefly, cells were incubated with EdU solution for 2 hours, fixed with 4% paraformaldehyde, and stained with Apollo dye and Hoechst 33342. Images were captured using a fluorescence microscope, and the percentage of EdU-positive cells was calculated to quantify cell proliferation.

### 2.18 Colony formation assay

LUAD cell lines were seeded into 6-well plates at a density of 500 cells per well and maintained under standard culture conditions. Cells were allowed to grow for approximately 10 days to enable the formation of visible colonies from single cells. At the end of the incubation period, colonies were fixed with 4% paraformaldehyde for 20 minutes at room temperature, followed by staining with 0.1% crystal violet solution for 15 minutes. Excess dye was gently washed off with PBS, and the plates were air-dried. Colonies consisting of more than 50 cells were counted manually under a microscope.

### 2.19 Wound healing assay

LUAD cell lines were seeded into 6-well plates and cultured until a confluent monolayer was formed. A linear wound was generated by gently scratching the cell monolayer with a sterile 200-μL pipette tip. Detached cells were removed by washing with PBS, and the medium was replaced with serum-free RPMI-1640 to suppress proliferation and facilitate directional migration. Images of the wound area were captured at 0, 24, and 48 hours post-scratch using an inverted microscope (Olympus IX81, Japan). The wound area at each time point was measured using ImageJ software (version 1.54g), and the percentage of wound closure was calculated using the following formula:

Healing percentage= (area of 0h –24h or 48h)/ (area of 0h).

### 2.20 Transwell migration assay

Cell migration ability was evaluated using a Transwell chamber assay. After appropriate treatment, 4 × 10^4^ LUAD cells were resuspended in serum-free medium and seeded into the upper chambers of Matrigel-coated Transwell inserts (Beyotime, Cat. No. C0371, Shanghai, China). The lower chambers were filled with medium supplemented with 10% fetal bovine serum (FBS), serving as a chemoattractant to facilitate cell migration through the porous membrane. After incubation for 24 hours at 37 °C, non-migrated cells on the upper surface of the membrane were carefully removed with a cotton swab. Cells that had traversed the membrane to the lower surface were fixed with pre-cooled 4% paraformaldehyde, then stained with 0.1% crystal violet. Migrated cells were visualized and imaged using an Olympus microscope, and the number of stained cells was quantified under randomly selected fields to assess migratory capacity.

### 2.21 *In vivo* tumorigenesis assay

Four-week-old male BALB/c nude mice were obtained from Beijing Huafukang Laboratory Animal Center (Beijing, China) and maintained under standard conditions. After one week of acclimatization, mice were subcutaneously injected with 5 × 10^6^ control or siRNA-transfected LUAD cells suspended in 100 μL of PBS. Tumor growth was monitored every 5 days by measuring tumor length and width with calipers, and tumor volume was calculated using the formula:


$$Volume=\frac{1}{2}\times length\times width^{2}$$

At day 25 post-injection, mice were euthanized under deep anesthesia with isoflurane followed by an overdose of pentobarbital sodium (150 mg/kg, i.p.). Tumors were then excised and weighed, and tumor size and weight were used as indicators to evaluate *in vivo* proliferative capacity. All animal experiments were performed in accordance with institutional guidelines and were approved by the Ethics Committee of the First Hospital of Jilin University (approval No. 20220015).

### 2.22 Statistical analysis

All statistical analyses were performed using R software (version 4.2.0). For comparisons between two independent groups, the Wilcoxon rank-sum test was employed, whereas the Kruskal–Wallis test was utilized for multi-group comparisons involving three or more groups. A two-sided p-value less than 0.05 was interpreted as indicative of statistical significance. The thresholds for significance were annotated as follows: p < 0.05 (), p < 0.01 (), and p < 0.001 ().

## 3 Results

### 3.1 Differential expression and functional enrichment of MRPGs in LUAD

**Figure 1:**
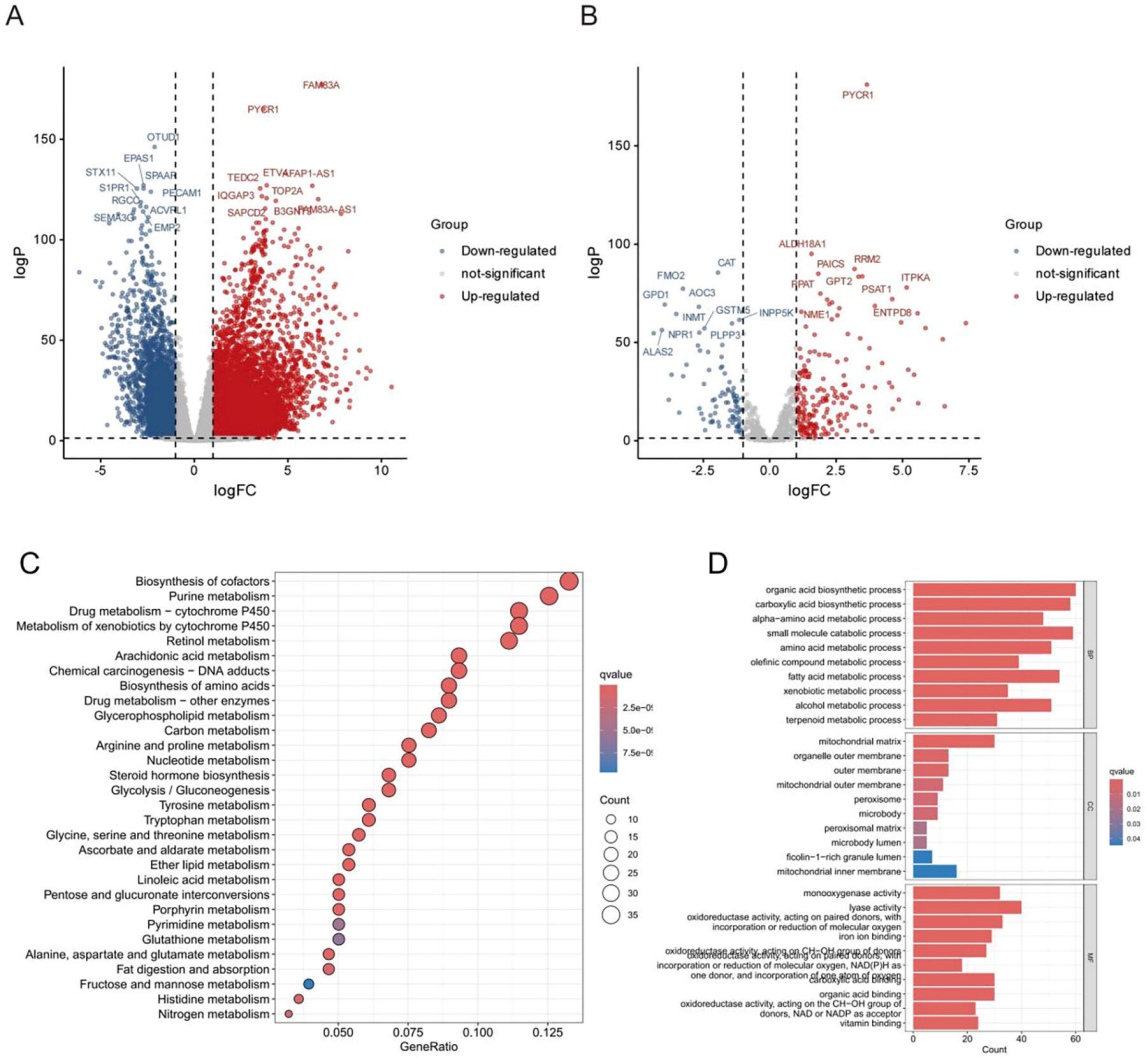
Differential expression and enrichment analysis of metabolic reprogramming-related genes between normal and LUAD tissues. **(A)** Volcano plot of all differentially expressed genes (DEGs) between normal lung tissues and LUAD tumor samples. Red dots: significantly upregulated genes; blue dots: significantly downregulated genes; gray dots: non-significant genes (adjusted p < 0.05, |log_2_FC| > 1). **(B)** Volcano plot showing differential expression of metabolic reprogramming-related genes (MRPGs) between normal and LUAD samples. Several MRPGs such as PYCR1, ALDH18A1, RRM2, and ITPKA were significantly upregulated in tumor tissues. **(C)** KEGG pathway enrichment analysis of upregulated MRPGs in LUAD, highlighting metabolic pathways including drug metabolism – cytochrome P450, retinol metabolism, and amino acid metabolism. **(D)** GO enrichment analysis of upregulated MRPGs categorized into Biological Process (BP), Cellular Component (CC), and Molecular Function (MF), revealing significant enrichment in mitochondrial functions, oxidoreductase activity, and organic acid biosynthesis.

To elucidate the molecular mechanisms underlying metabolic reprogramming in lung adenocarcinoma (LUAD), we first performed a transcriptome-wide differential expression analysis between tumor and normal lung tissues. This analysis revealed widespread transcriptional alterations, with numerous genes significantly upregulated or downregulated in LUAD (**Figure 1A**), indicating a substantial reshaping of the gene expression landscape. We then focused on metabolic reprogramming-related genes (MRPGs) and compared their expression profiles between normal and tumor tissues. Several MRPGs exhibited significant dysregulation in LUAD samples (**Figure 1B**), suggesting a strong association between metabolic alterations and transcriptional regulation. To investigate the functional significance of these dysregulated MRPGs, we conducted KEGG and Gene Ontology (GO) enrichment analyses. KEGG analysis showed that these genes were significantly enriched in purine metabolism, cytochrome P450-mediated drug metabolism, and oxidative phosphorylation pathways (**Figure 1C**, **Supplementary Figure S1G**), implicating their involvement in energy production and metabolic detoxification. GO analysis further revealed enrichment in biological processes associated with mitochondrial function, organic acid biosynthesis, and oxidoreductase activity (**Figure 1D**, **Supplementary Figure S1H**), highlighting the central role of MRPGs in maintaining cellular redox homeostasis and metabolic integrity. Taken together, these findings demonstrate a global transcriptional reprogramming of MRPGs in LUAD, emphasizing their critical involvement in mitochondrial regulation and metabolic adaptation. These results provide mechanistic insights into LUAD metabolic dysregulation and offer a potential molecular basis for developing metabolism-oriented therapeutic strategies.

### 3.2 Construction and validation of the metabolic reprogramming-based prognostic signature

**Figure 2:**
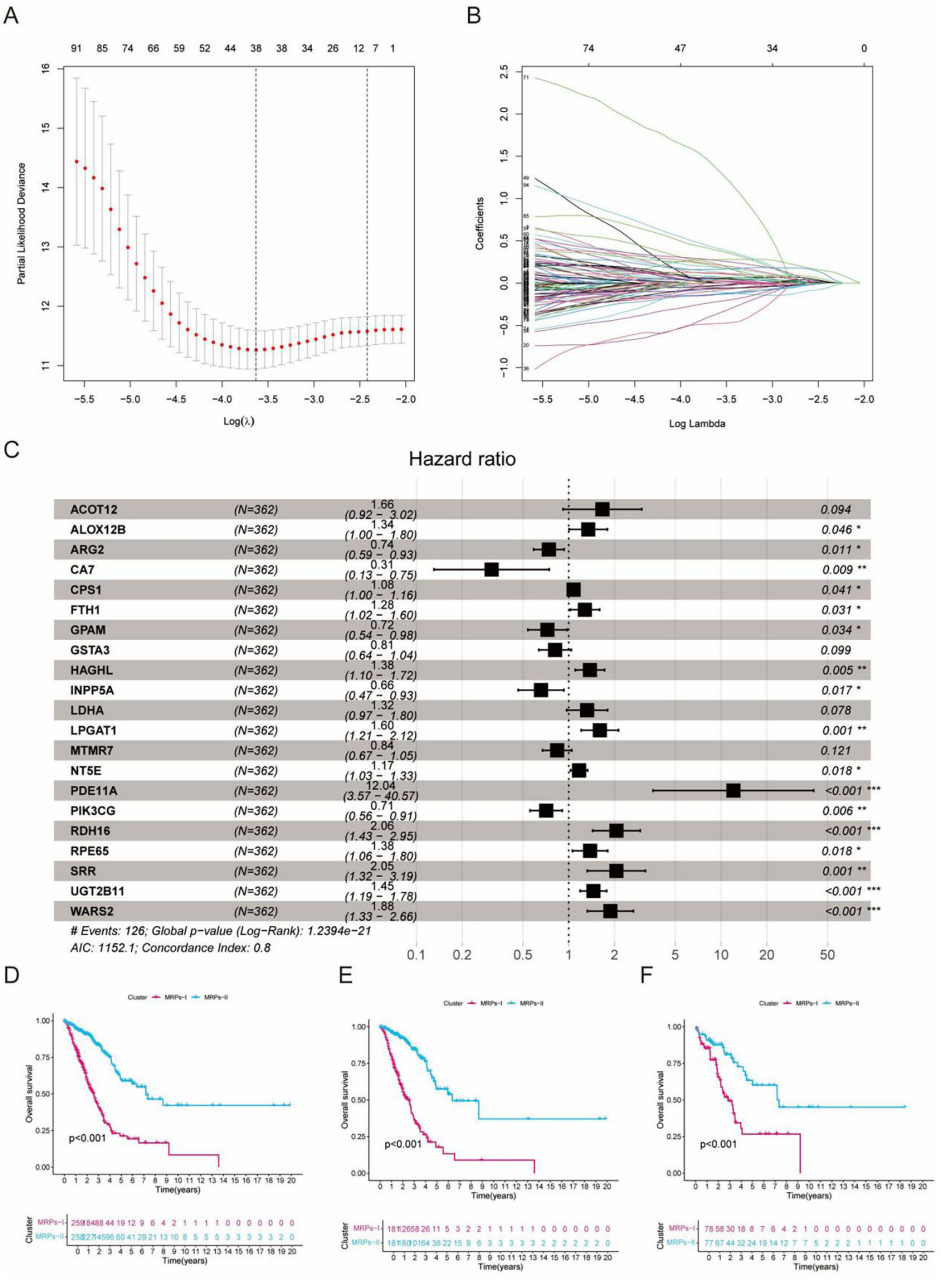
Construction and validation of the metabolic reprogramming-based prognostic score (MRPs) model in LUAD using TCGA cohort. **(A)** Ten-fold cross-validation for tuning parameter selection in the LASSO regression model. **(B)** LASSO coefficient profiles of the 21 candidate metabolic reprogramming-related genes. **(C)** Forest plot of multivariate Cox regression analysis for the selected genes. Several genes. **(D–F)** Kaplan–Meier survival curves comparing overall survival between MRP-I and MRP-II subgroups in the entire TCGA cohort **(D)**, TCGA training set **(E)**, and TCGA testing set **(F)**. MRP-I subgroup exhibits significantly worse prognosis in all datasets (log-rank p < 0.001). *P<0.05, **P<0.01, ***P<0.001.

To develop a robust prognostic model grounded in metabolic reprogramming-related genes (MRPGs), we employed least absolute shrinkage and selection operator (LASSO) Cox regression to perform feature selection and prevent model overfitting. The optimal regularization parameter (λ) was determined *via* 10-fold cross-validation, resulting in the retention of 21 MRPGs with the highest prognostic relevance (**Figures 2A, B**). Subsequent multivariate Cox regression analysis confirmed that a majority of these genes were significantly associated with overall survival, with several acting as strong protective or risk factors (**Figure 2C**). Using the regression coefficients derived from the final model, we calculated a metabolic reprogramming score (MRPs) for each patient. Based on the median MRPs value, patients were stratified into two distinct metabolic subtypes: MRP I and MRP II. Kaplan–Meier survival analysis revealed that patients in the MRP I group exhibited significantly worse overall survival compared to those in the MRP II group, consistently observed in the entire TCGA-LUAD cohort (**Figure 2D**), as well as in both training (**Figure 2E**) and validation subsets (**Figure 2F**), underscoring the robust prognostic stratification power of this signature. To further examine the expression profiles of the 21 model genes, we compared their transcript levels between the MRP I and MRP II groups. As illustrated in the violin plots (**Supplementary Figure S2**), a large proportion of MRPGs exhibited significant differential expression between high-risk and low-risk patients, providing additional evidence for their potential involvement in LUAD metabolic heterogeneity. Notably, genes such as LDHA, ARG2, SRR, and WARS2 showed particularly strong expression differences, further reinforcing their relevance in risk stratification. In addition, we evaluated the prognostic value of individual model genes by performing Kaplan–Meier survival analyses for both overall survival (OS) and disease-free survival (DFS). Several genes, including RDH16, WARS2, and to a lesser extent PDE11A, demonstrated distinct survival associations (**Supplementary Figure S3**). Among them, WARS2 stood out due to its significant correlation with poor OS (p = 0.0072, HR = 1.8) and a marginal association with DFS (p = 0.057), suggesting it may serve as a critical prognostic marker in LUAD. Given its strong differential expression pattern and pronounced impact on survival, we selected WARS2 for subsequent in-depth functional investigation. Follow-up analyses focused on characterizing its biological roles in LUAD progression, including *in vitro* validation experiments to elucidate its mechanistic contribution to metabolic reprogramming and tumor aggressiveness.

### 3.3 Independent prognostic value and predictive performance of the MRPs signature

**Figure 3:**
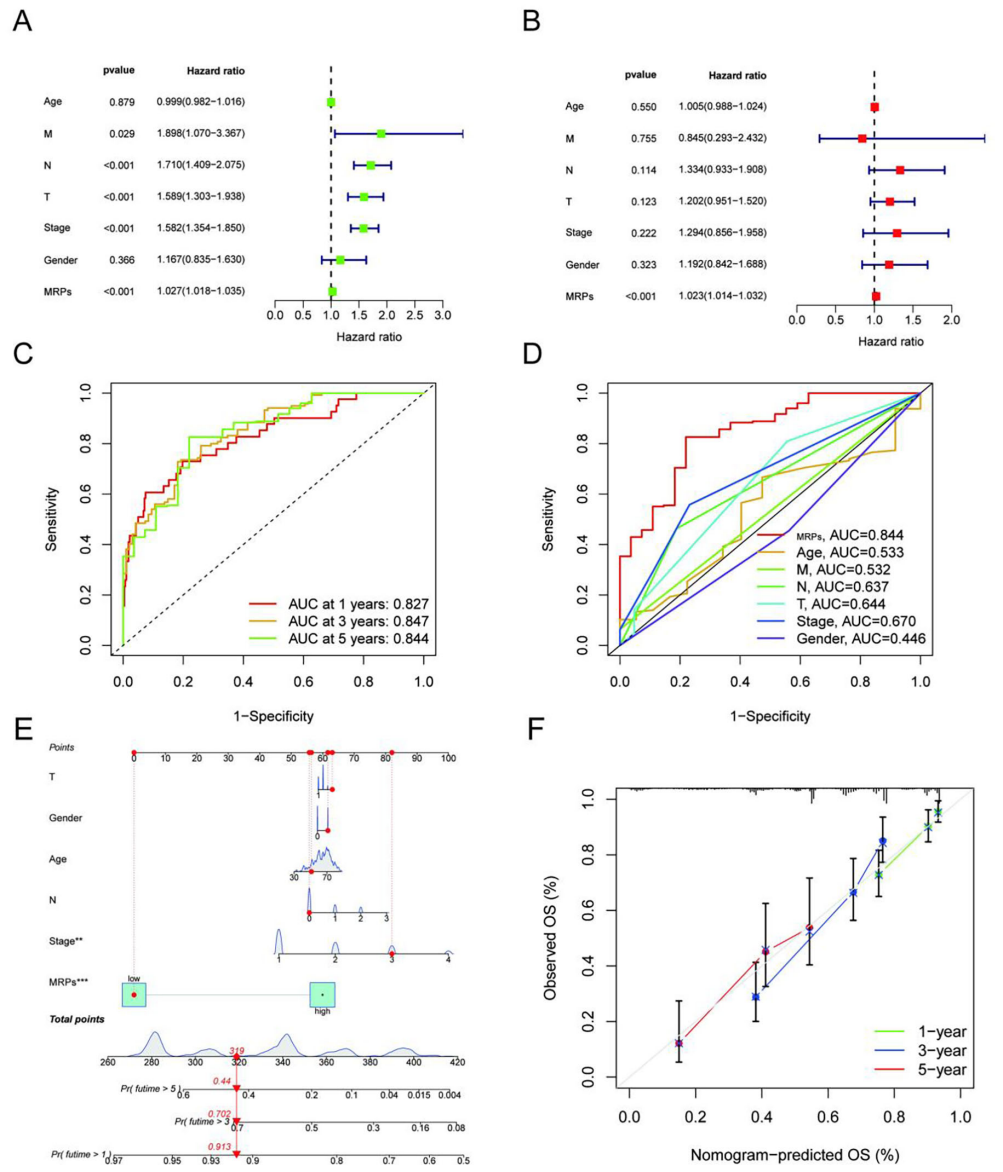
Development and independent validation of a MRPs-prognostic model for LUAD: a nomogram with robust predictive performance in training and testing cohorts. **(A, B)** Univariate **(A)** and multivariate **(B)** Cox regression analyses in the TCGA training cohort demonstrate that the MRPs score is an independent prognostic factor for overall survival after adjusting for age, sex, TNM stage, and other clinical variables. **(C)** Time-dependent ROC curves show the high predictive accuracy of the MRPs score for 1-, 3-, and 5-year overall survival in the training cohort (AUC = 0.827, 0.847, and 0.844, respectively). **(D)** ROC comparison indicates that the MRPs score outperforms conventional clinical parameters (age, M, N, T, stage, and gender) in predicting survival outcomes in the training set. **(E)** A prognostic nomogram was developed based on the TCGA LUAD training cohort by incorporating MRPs score along with clinical features including T stage, N stage, age, gender, and overall stage. Each variable is assigned a score on the point scale, and the total score corresponds to the estimated probability of 1-, 3-, and 5-year overall survival (OS). **(F)** Calibration plots for 1-, 3-, and 5-year OS show good agreement between nomogram-predicted and actual survival outcomes, indicating reliable predictive performance of the model. *P<0.05, **P<0.01, ***P<0.001.

To determine whether the MRPs signature functions as an independent prognostic factor in LUAD, we conducted both univariate and multivariate Cox regression analyses. In the TCGA-LUAD cohort, MRPs remained significantly associated with overall survival in both univariate (**Figure 3A**) and multivariate models (**Figure 3B**), independent of other clinical parameters including age, gender, and TNM stage. Similar findings were observed in the training (**Supplementary Figures S4D, E**) and testing subsets, confirming the robustness and reproducibility of the model across different cohorts. We further assessed the predictive performance of the MRPs signature using time-dependent receiver operating characteristic (ROC) curves. In the overall TCGA-LUAD cohort, the area under the curve (AUC) for 1-, 3-, and 5-year survival was 0.827, 0.847, and 0.844, respectively (**Figure 3D**), indicating excellent prognostic accuracy. Notably, when compared with conventional clinical variables, the MRPs signature consistently outperformed age, gender, and TNM components, achieving the highest AUC (0.844) among all factors (**Figure 3E**). This superior performance was further validated in both the training and testing subsets, where the MRPs signature demonstrated strong time-dependent AUC values and exhibited significantly higher predictive power relative to traditional clinical indicators (**Supplementary Figure S4F, G**). Collectively, these findings underscore the independent prognostic value and robust discriminative ability of the MRPs-based model in stratifying LUAD patient outcomes.

### 3.4 Construction and validation of a prognostic nomogram based on MRPs

To further translate the MRPs signature into a clinically applicable tool, we constructed a prognostic nomogram incorporating MRPs classification along with key clinical variables, including T stage, N stage, age, gender, and pathological stage (**Figure 3E**). Each variable was assigned a weighted point score, and the cumulative score corresponded to the estimated probabilities of 1-, 3-, and 5-year overall survival (OS). Notably, the MRPs classification emerged as one of the most influential components in the nomogram, reflecting its substantial contribution to risk stratification. To evaluate the predictive accuracy of the nomogram, we performed calibration curve analysis. The predicted survival probabilities showed excellent concordance with the observed outcomes at 1, 3, and 5 years (**Figure 3F**), indicating that the model is well-calibrated. These results suggest that the MRPs-integrated nomogram provides a reliable and individualized prognostic assessment for LUAD patients, with strong potential for clinical implementation in routine decision-making.

### 3.5 Differential gene expression and functional enrichment between MRPs I and II subgroups

To elucidate the biological differences underlying distinct metabolic risk states, we performed transcriptome-wide differential expression analysis between the MRP I and MRP II subgroups. A substantial number of differentially expressed genes (DEGs) were identified across the whole transcriptome (**Supplementary Figure S1A**), alongside focused analysis of differentially expressed metabolic reprogramming-related genes (MRPGs) (**Supplementary Figure S1B**). These results revealed extensive transcriptional reprogramming associated with MRPs classification. Functional enrichment analysis of global DEGs indicated significant involvement in pathways such as xenobiotic metabolism by cytochrome P450, retinol metabolism, chemical carcinogenesis, and immune-related processes, as revealed by KEGG and Gene Ontology (GO) analyses (**Supplementary Figure S1C, D**). These findings suggest that both metabolic and immune alterations distinguish patients between the two MRPs subgroups. To gain deeper insight into the metabolic underpinnings, we performed functional enrichment on the subset of differentially expressed MRPGs. KEGG analysis highlighted consistent enrichment in core metabolic circuits, including drug metabolism, porphyrin metabolism, and lipid-associated pathways (**Supplementary Figure S1E**). GO analysis further revealed overrepresentation of biological processes related to hormone metabolism, oxidoreductase activity, and mitochondrial components (**Supplementary Figure S1F**), underscoring the critical role of metabolic rewiring in shaping the distinct molecular states of MRP I and MRP II patients.

### 3.6 Pathway enrichment analysis reveals distinct functional states between MRPs subgroups

**Figure 4:**
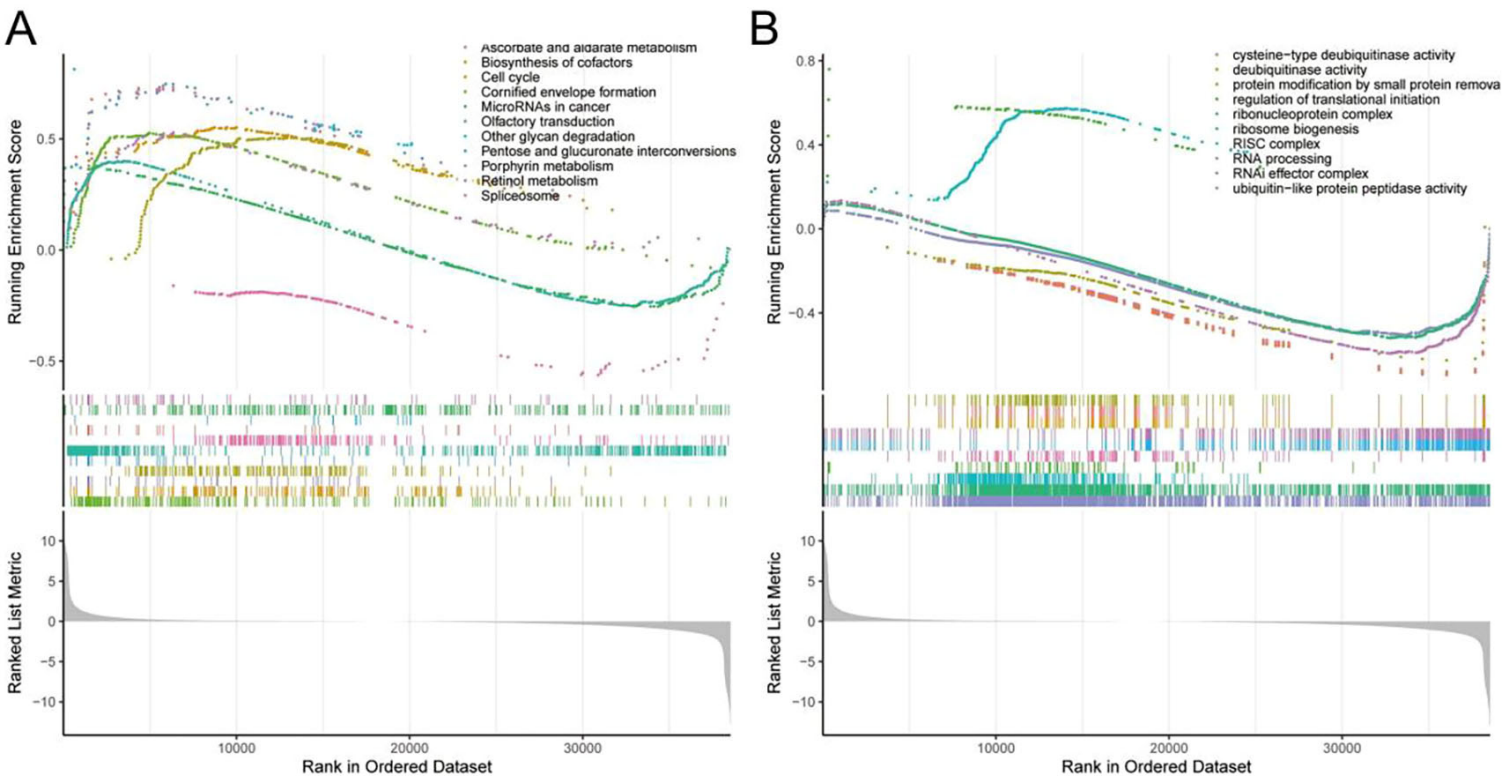
GSEA analysis reveals distinct functional signatures between MRP I and MRP II subgroups in LUAD. **(A)** Gene Set Enrichment Analysis (GSEA) based on KEGG terms between MRP I and MRP II subgroups. Representative upregulated pathways in MRP I included retinol metabolism, cytochrome P450 detoxification, and drug metabolism. **(B)** GSEA based on GO Molecular Function terms, showing enrichment of enzyme activity–related terms such as oxidoreductase and ubiquitin-like protein ligase binding in MRP I subgroup.

To further elucidate the biological pathways underlying metabolic risk stratification, we performed gene set variation analysis (GSVA) and gene set enrichment analysis (GSEA) between the MRP I and MRP II subgroups. GSVA was conducted using KEGG and GO gene sets to assess pathway activity at the sample level. As shown in the KEGG-based heatmap (**Supplementary Figure S5A**), MRP I tumors exhibited higher activity in several metabolic and signaling pathways compared to MRP II, including pyrimidine metabolism, purine metabolism, mismatch repair, and the cell cycle. Similarly, the GO-based GSVA results (**Supplementary Figure S5B**) demonstrated elevated activation of biological processes associated with DNA replication, chromatin organization, and RNA metabolic regulation in the MRP I group, indicating heightened proliferative and transcriptional activity. GSEA further supported these observations. In the Biological Process (BP) category (**Figure 4A**), MRP I tumors were significantly enriched in pathways such as chromosome segregation, DNA replication, and mitotic spindle assembly. Moreover, the Molecular Function (MF) category analysis (**Figure 4B**) highlighted upregulation of pathways involved in ubiquitin-like protein ligase activity, helicase activity, and ribosomal structural components. Collectively, these findings suggest that tumors in the MRP I subgroup are characterized by enhanced cell cycle progression, nucleic acid metabolism, and proteostasis mechanisms—hallmarks often associated with more aggressive tumor phenotypes.

### 3.7 Immune microenvironment characteristics associated with MRPs and WARS2 expression

**Figure 5:**
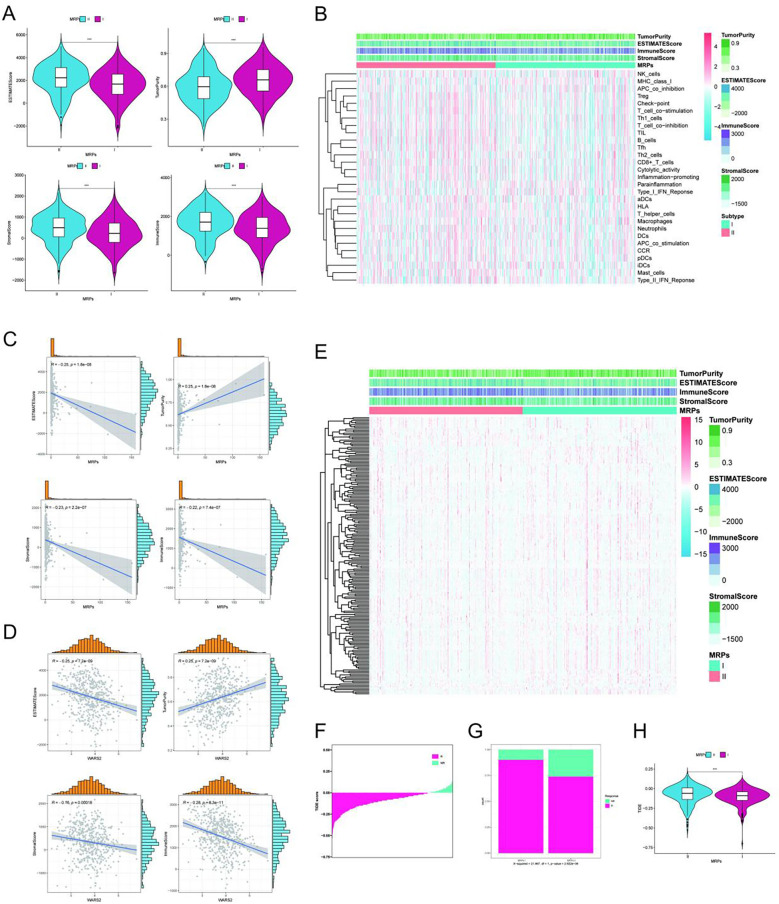
Associations between MRPs subtypes, immune microenvironment, immunotherapy, and WARS2 expression in LUAD. **(A)** Violin plots comparing ESTIMATEScore, TumorPurity, StromalScore, and ImmuneScore between MRP I and MRP II subgroups. MRP II samples exhibited significantly lower ESTIMATEScore, StromalScore, and ImmuneScore, and higher tumor purity (all p < 0.001). **(B)** Heatmap showing the relative abundance of immune-related functional modules and immune cell subsets across MRPs subtypes, suggesting distinct immune landscape between MRP I and MRP II. **(C)** Correlation between MRP score and ESTIMATEScore, TumorPurity, StromalScore, and ImmuneScore, indicating that higher MRP scores are associated with a more immune-depleted tumor microenvironment. **(D)** Correlation between WARS2 expression and ESTIMATEScore, TumorPurity, StromalScore, and ImmuneScore, showing that high WARS2 expression is similarly linked to lower immune and stromal content and increased tumor purity. **(E)** Heatmap of predicted drug sensitivity (estimated IC50 values) for anti-tumor compounds across TCGA LUAD samples. Columns represent patients, rows represent compounds; hierarchical clustering is shown. Top annotation bars indicate TumorPurity, ESTIMATEScore, ImmuneScore, StromalScore, and MRPs subtype (I vs II), highlighting subtype-associated pharmacologic patterns. **(F)** Waterfall plot of TIDE scores for individual patients, ordered from low to high. Bars are colored by predicted immune checkpoint blockade **(ICB)** response status (R, responder; NR, non-responder). **(G)** Stacked bar plot summarizing the proportion of predicted ICB responders in MRP I vs MRP II subgroups (χ² test p value shown). MRP I harbors a higher fraction of predicted responders. **(H)** Violin plot comparing TIDE scores between MRPs subgroups. MRP I shows significantly lower TIDE scores (**p < 0.001), suggesting reduced immune evasion potential and greater likelihood of benefit from ICB therapy. *P<0.05, **P<0.01, ***P<0.001.

To explore the relationship between metabolic risk and the tumor immune microenvironment in LUAD, we first employed the ESTIMATE algorithm to quantify stromal and immune components across samples. Compared to the MRPs II group, patients in the MRPs I subgroup exhibited significantly lower ImmuneScore, StromalScore, and ESTIMATEScore, but higher TumorPurity (**Figure 5A**), suggesting a less infiltrated and more immune-desert tumor microenvironment in the high-risk group. To further dissect immune landscape differences, we performed single-sample gene set enrichment analysis (ssGSEA) using 29 immune-related gene signatures. As shown in the heatmap (**Figure 5B**), the MRPs II subgroup exhibited broader and stronger enrichment of multiple immune cell populations and immune-related pathways, including CD8^+^ T cells, regulatory T cells (Tregs), macrophages, dendritic cells, and several inflammation-associated responses—indicative of a more active immune contexture compared to MRPs I tumors. Correlation analysis further confirmed these associations. Spearman correlation revealed that MRPs was negatively correlated with ImmuneScore (R = –0.26), StromalScore (R = –0.23), and ESTIMATEScore (R = –0.26), but positively correlated with TumorPurity (R = 0.25), all statistically significant (**Figure 5C**), reinforcing the notion that higher MRPs scores are linked to an immunologically "cold" tumor phenotype. Strikingly, WARS2, one of the most prognostically significant genes in our model, showed a parallel trend. Its expression was positively correlated with TumorPurity (R = 0.25) and negatively correlated with stromal and immune infiltration metrics (**Figure 5D**), suggesting that WARS2 may contribute to the establishment or maintenance of an immune-excluded microenvironment. Together, these results demonstrate that elevated metabolic risk, as indicated by high MRPs scores and WARS2 expression, is associated with reduced immune infiltration and an immunosuppressive tumor milieu—potentially underlying the poor prognosis observed in the MRP I subgroup.

### 3.8 Mutation landscape and tumor mutation burden in relation to MRPs stratification

To investigate the somatic mutational landscape of LUAD and its association with metabolic risk stratification, we analyzed the mutation profiles derived from TCGA. Waterfall plots illustrated the top 20 most frequently mutated genes in the entire cohort (**Supplementary Figure S6A**) as well as within the MRPG set (**Supplementary Figure S6B**). Consistent with known LUAD mutational patterns, TP53, TTN, and MUC16 exhibited the highest mutation frequencies, reaffirming their roles as common drivers in lung adenocarcinoma. To further explore genomic instability, we calculated tumor mutation burden (TMB) for each sample. The distribution of TMB across the cohort is shown in **Supplementary Figure S6C**. Interestingly, no significant difference in TMB was observed between the MRPs I and MRPs II subgroups (**Supplementary Figure S6D**), suggesting that MRPs-defined transcriptional and immune differences may occur independently of total mutational load. This observation supports the hypothesis that metabolic reprogramming may influence tumor aggressiveness and immune evasion through non-genomic mechanisms—such as metabolic-immune crosstalk, altered signaling pathways, or epigenetic regulation—rather than *via* increased mutation burden per se. Comprehensive mutational analysis further revealed the predominance of missense mutations and a high frequency of C>T transitions across the LUAD cohort (**Supplementary Figure S6E, F**). Both MRP subgroups exhibited similar patterns in overall mutation classification and single-nucleotide variant (SNV) spectra, reinforcing the conclusion that the transcriptional and metabolic risks captured by the MRPs signature are largely independent of mutational burden or subtype.

### 3.9 Drug sensitivity and immunotherapy response analysis based on MRPs classification

To investigate the therapeutic implications of metabolic risk stratification, we performed drug sensitivity and immunotherapy response analyses between MRPs-defined subgroups. Drug response profiling revealed distinct pharmacological sensitivities between MRP I and MRP II tumors. The IC50-based heatmap indicated that multiple compounds exhibited significantly different predicted sensitivities across the two groups, suggesting unique metabolic dependencies and the potential for tailored treatment strategies (**Figure 5E**). To assess immune evasion potential, we applied the Tumor Immune Dysfunction and Exclusion (TIDE) algorithm. The TIDE score distribution revealed that the majority of MRP I tumors harbored lower TIDE values (**Figure 5F**), implying reduced immune escape and a higher likelihood of benefit from immune checkpoint blockade (ICB). Supporting this, the bar plot showed a significantly greater proportion of predicted ICB responders in the MRP I group compared to MRP II (**Figure 5G**). Furthermore, the overall TIDE score was markedly lower in MRP I patients, as shown in the violin plot (**Figure 5H**). These results suggest that although MRP I tumors represent a metabolically high-risk group, they may paradoxically retain greater immunotherapy sensitivity, potentially due to less severe immune dysfunction or exclusion. This highlights a complex interplay between metabolic reprogramming and immune responsiveness and suggests that MRP I patients could particularly benefit from immune checkpoint-based interventions.

### 3.10 Single-cell transcriptomic profiling reveals cell-type-specific expression and functional implications of WARS2

**Figure 6:**
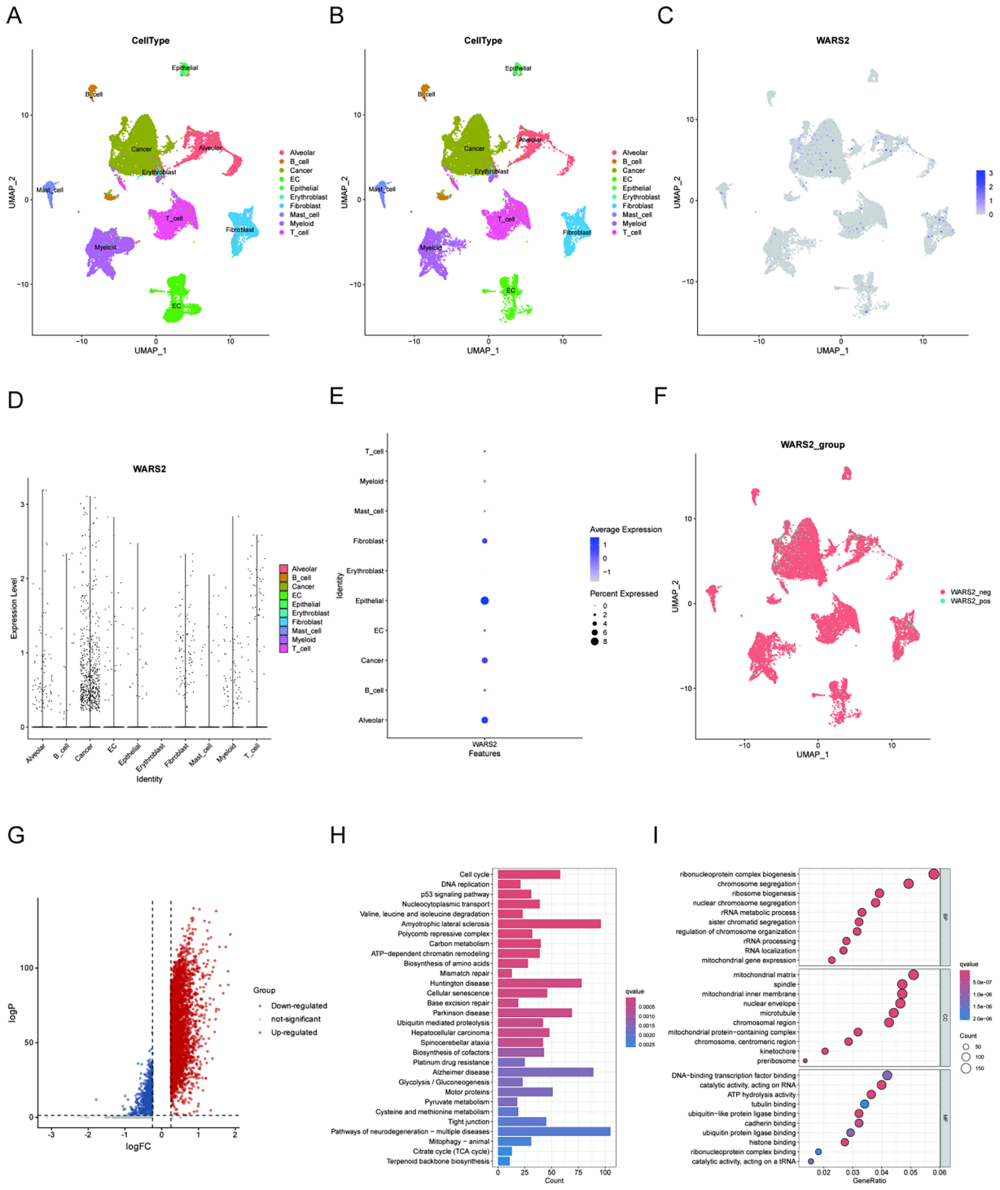
Single-cell transcriptomic landscape of WARS2 expression and its associated transcriptional features in LUAD. **(A, B)** UMAP plots showing cell type annotations for all cells **(A)** and tumor-derived cells only **(B)** from LUAD single-cell RNA-seq datasets. **(C)** Feature plot depicting WARS2 expression levels across all cells. WARS2 is predominantly expressed in cancer cells. **(D)** Violin plot comparing WARS2 expression across major cell types. Cancer cells display the highest expression levels. **(E)** Dot plot summarizing the expression distribution of WARS2 among different cell types; dot size represents percentage of expressing cells, and color intensity indicates average expression. **(F)** UMAP plot showing classification of cancer cells into WARS2-positive (WARS2_pos) and WARS2-negative (WARS2_neg) groups based on non-zero expression threshold. **(G)** Volcano plot of differentially expressed genes between WARS2_pos and WARS2_neg cancer cells. Red and blue dots represent significantly up- and down-regulated genes, respectively. **(H, I)** Bar plot **(H)** and bubble plot **(I)** showing GO enrichment results of differentially expressed genes between WARS2_pos and WARS2_neg cancer cells, highlighting biological processes such as cell cycle regulation, mitochondrial function, and protein translation.

To delineate the cell-type-specific expression pattern and biological function of WARS2 in lung adenocarcinoma (LUAD), we performed single-cell RNA sequencing analysis across tumor and normal lung tissues. UMAP projection of all captured cells revealed clear segregation of major cell populations, including epithelial cells, immune cells, fibroblasts, endothelial cells, and myeloid lineages (**Figure 6A**). We then restricted the analysis to cells derived specifically from tumor tissues to better capture cancer-relevant transcriptional features (**Figure 6B**). Within the tumor microenvironment, WARS2 expression was predominantly enriched in malignant epithelial (cancer) cells, with minor expression observed in fibroblasts and endothelial cells, and minimal expression across immune cell types (**Figures 6C–E**). Based on WARS2 expression status, we further stratified tumor-derived epithelial cells into WARS2-positive and WARS2-negative subpopulations for downstream comparative analysis (**Figure 6F**). Differential expression analysis between WARS2^+^ and WARS2^-^ cancer cells revealed widespread transcriptional reprogramming (**Figure 6G**). Genes upregulated in WARS2^+^ cells were significantly enriched in pathways related to cell cycle regulation, oxidative phosphorylation, and ribosomal function, suggesting enhanced metabolic and proliferative activity. Functional enrichment analysis further supported these findings. KEGG pathway analysis highlighted activation of DNA replication, cell cycle, oxidative phosphorylation, and ribosome biogenesis (**Figure 6H**), while GO enrichment underscored biological processes such as mitochondrial protein import, RNA processing, and translation initiation (**Figure 6I**). Together, these results indicate that WARS2 is specifically overexpressed in LUAD cancer cells, where it marks a transcriptional state characterized by elevated biosynthetic and proliferative programs. This tumor-specific and metabolically active signature supports WARS2 as a potential functional driver and therapeutic target in LUAD.

### 3.11 WARS2^+^ cancer cells exhibit enhanced functional activity and distinct communication networks

**Figure 7:**
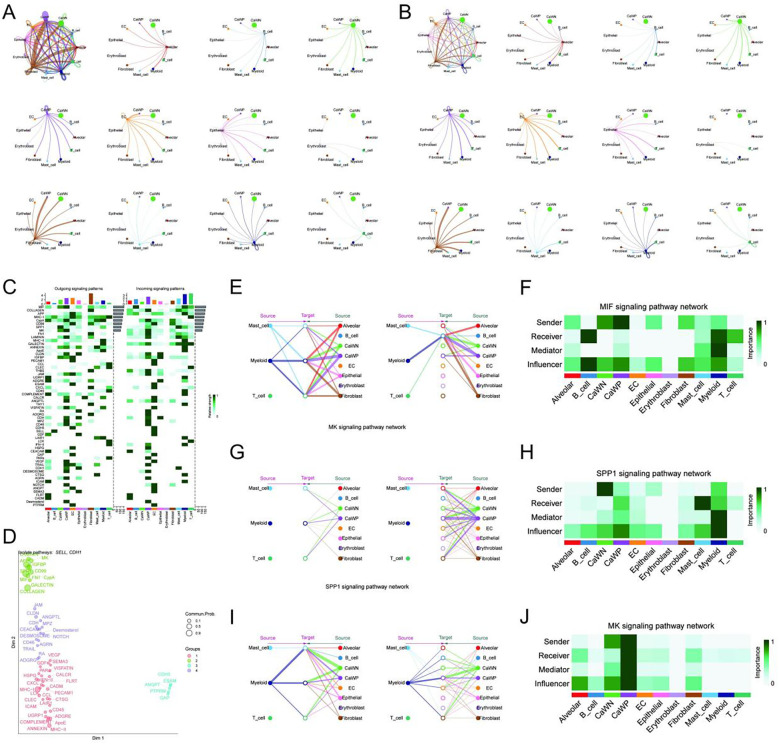
Quantitative analysis of cell–cell communication patterns associated with WARS2 expression in LUAD. **(A)** Overview of inferred intercellular communication networks for WARS2-positive (CaWP) and WARS2-negative (CaWN) cancer cells. The leftmost circle plot displays the global signaling interactions, while the following plots illustrate the outgoing signaling networks from CaWP and CaWN cells through representative signaling pathways including COLLAGEN, FN1, CD99, MIF, LAMININ, VISFATIN, and others. Each edge represents a predicted ligand-receptor interaction, with thickness proportional to interaction strength. **(B)** Incoming signaling patterns targeting CaWP and CaWN cells from surrounding cell types. The initial circle plot shows global incoming communication, and subsequent panels depict contributions from individual signaling pathways. These results highlight distinct interaction landscapes between WARS2-expressing and non-expressing cancer cells, suggesting a role for WARS2 in modulating tumor microenvironmental crosstalk. **(C)** Heatmap displaying outgoing (left) and incoming (right) signaling strength across different cell types for major ligand–receptor signaling pathways, including MIF, MK, SPP1, COLLAGEN, CD99, FN1, and others. CaWP: WARS2-positive cancer cells; CaWN: WARS2-negative cancer cells. **(D)** Network embedding of ligand–receptor pairs using manifold learning to identify pathway clusters. Pathways related to structural components and inflammation (e.g., MIF, FN1, GALLECTIN) formed distinct functional modules. **(E, F)** MIF signaling network: **(E)** Edge plots depict directional communication between sender and receiver cell types for WARS2-high and WARS2-low groups. **(F)** Heatmap quantifies sender, receiver, mediator, and influencer roles across cell types. **(G, H)** MK signaling network: **(G)** Interaction diagrams for WARS2 groups; **(H)** Cell-type roles in the MK signaling axis. **(I–J)** SPP1 signaling network: **(I)** Source–target relationships; **(J)** Functional importance heatmap. These results demonstrate that WARS2-positive tumor cells are embedded in a distinct signaling microenvironment, particularly enriched for immunomodulatory and matrix-associated communication pathways.

To further elucidate the functional roles of WARS2-positive cancer cells in LUAD, we performed AUCell-based pathway activity scoring and CellChat-mediated intercellular communication analysis. AUCell results showed that WARS2^+^ cancer cells (CaWP) exhibited elevated activity in multiple biological pathways compared to their WARS2^-^ counterparts (CaWN) (**Supplement Table 1**). Among the most significantly enriched gene sets were those involved in oxidative stress response (e.g., peroxiredoxin activity, thioredoxin-dependent peroxiredoxin activity), RNA processing (e.g., 7-methylguanosine cap hypermethylation), protein synthesis, and mitochondrial function. These findings suggest that WARS2^+^ cells are characterized by enhanced redox homeostasis, transcriptional efficiency, and mitochondrial biosynthetic programs—hallmarks of metabolically active and proliferative cancer phenotypes. To investigate how WARS2 expression affects cellular communication, we applied CellChat analysis to compare interaction patterns between CaWP and CaWN groups. In both the interaction count (**Figure 7A**) and interaction weight (**Figure 7B**) networks, CaWP cells demonstrated a denser and more extensive communication architecture. Notably, CaWP cells sent significantly more signals to endothelial cells, fibroblasts, and myeloid populations, implicating these cells in modulating the tumor microenvironment. Pathway-level breakdown revealed that CaWP cells exhibited enhanced outgoing signaling *via* COLLAGEN, FN1, LAMININ, and CD44 axes—key mediators of extracellular matrix organization and tumor–stroma interaction. This suggests that WARS2^+^ cells not only possess higher metabolic and transcriptional activity, but also play a central role in coordinating microenvironmental remodeling and potentially immune evasion. Collectively, these data highlight the dual role of WARS2^+^ LUAD cancer cells as metabolically reprogrammed and communication-competent entities, capable of shaping both intracellular and intercellular tumor biology.

### 3.12 WARS2^+^ cancer cells are central hubs in key intercellular communication pathways

To further elucidate how WARS2-positive cancer cells (CaWP) integrate into and influence the tumor microenvironment, we conducted a comprehensive pathway-level cell-cell communication analysis using the CellChat framework. Global signaling activity analysis revealed that CaWP cells exhibited markedly enhanced outgoing and incoming communication compared to WARS2-negative cancer cells (CaWN), suggesting that WARS2 expression is associated with a more interactive and functionally engaged cellular state (**Figure 7C**). Clustering of signaling pathways based on their pattern similarity further identified distinct communication modules, with key axes such as MIF, MK, and SPP1 emerging as major hubs within the CaWP-centric network (**Figure 7D**). Focusing on these top pathways, we found that CaWP cells played a prominent role in the MIF signaling network, participating as both major signal senders and recipients in interactions with immune cells, endothelial cells, and fibroblasts (**Figure 7E**). Quantitative role analysis confirmed that CaWP cells acted as influential mediators and initiators within this pathway, implicating them in immune modulation and inflammatory signaling (**Figure 7F**). A similar pattern was observed for MK signaling, where CaWP cells transmitted strong signals particularly toward stromal components, including fibroblasts and endothelial cells (**Figure 7G**), and were quantitatively ranked as primary receivers, mediators, and influencers (**Figure 7J**), consistent with their involvement in growth factor-mediated tumor–stroma communication. Within the SPP1 signaling axis, CaWP cells again displayed widespread involvement, establishing extensive bidirectional signaling with various microenvironmental components (**Figure 7I**). They were especially dominant as senders and influencers (**Figure 7H**), highlighting a potential role in extracellular matrix remodeling and immune cell recruitment. Together, these results reveal that WARS2-positive cancer cells are not only metabolically and transcriptionally active but also occupy central positions in the LUAD intercellular signaling landscape. Their prominent roles across multiple pro-tumorigenic communication pathways reinforce the hypothesis that WARS2 overexpression contributes to tumor progression through both cell-intrinsic metabolic rewiring and cell-extrinsic microenvironmental modulation.

### 3.13 Functional and mechanistic validation of WARS2 in LUAD

**Figure 8:**
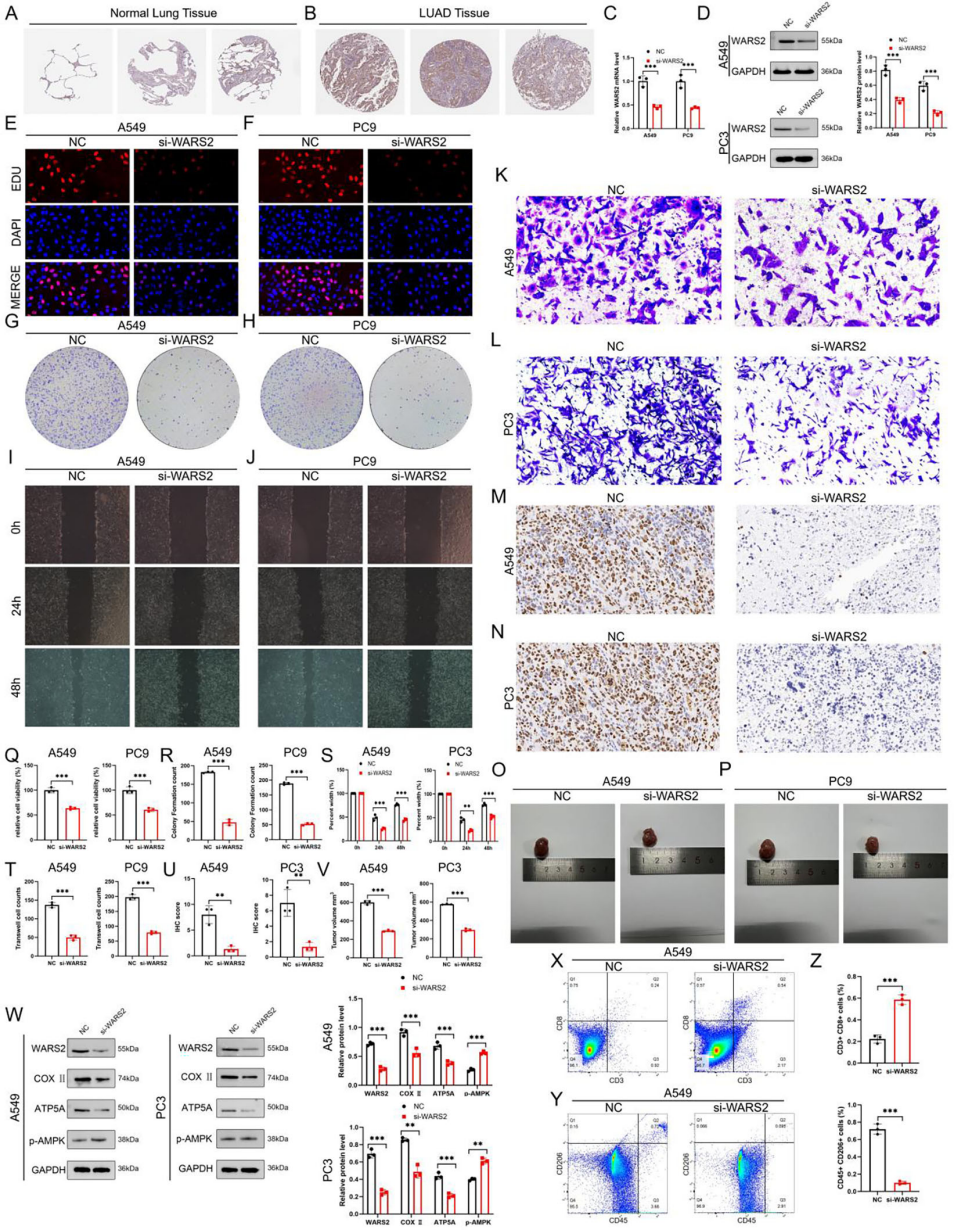
Functional and mechanistic validation of WARS2 in LUAD. **(A, B)** Immunohistochemical staining of WARS2 in **(A)** normal lung tissues and **(B)** LUAD tissues. WARS2 protein showed strong positive staining in tumor sections compared to adjacent normal tissue. **(C, D)** Validation of WARS2 knockdown efficiency in A549 and PC9 cells using RT-qPCR and Western blotting. **(E, F)** EdU incorporation assays showing decreased proliferation of A549 and PC9 cells after WARS2 silencing. **(G, H)** Colony formation assays confirming reduced clonogenic ability upon WARS2 knockdown. **(I, J)** Wound healing assays demonstrating impaired migratory ability in si-WARS2 cells compared with controls at 0 h and 48 (h) **(K, L)** Transwell invasion assays showing decreased invasive capacity after WARS2 depletion. **(M, N)** Immunohistochemical staining of xenograft tumors derived from A549 and PC9 cells, confirming reduced WARS2 protein expression in the si-WARS2 group. **(O, P)** Representative images of xenograft tumors from nude mice injected with si-NC or si-WARS2 A549 and PC9 cells, indicating reduced tumor size after WARS2 knockdown. **(Q, V)** Quantitative analyses of cell proliferation, migration, and invasion based on EdU, colony formation, wound healing, and Transwell assays in A549 and PC9 cells. **(W)** Western blot analysis of mitochondrial oxidative phosphorylation (OXPHOS) markers (COX I, ATP5A) and energy stress sensor (p-AMPK) in si-WARS2 and control cells; densitometric quantification is shown on the right. **(X, Y)** Flow cytometric analysis of tumor-infiltrating immune cells from A549-derived xenografts, demonstrating an increase in CD3^+^CD8^+^ cytotoxic T cells and a decrease in CD45^+^CD206^+^ M2-like macrophages after WARS2 silencing. **(Z)** Quantification of CD8^+^ T cells and M2 macrophages from **(X, Y)**, indicating a significant shift toward an immune-active microenvironment in the si-WARS2 group. *P<0.05, **P<0.01, ***P<0.001.

To experimentally verify the oncogenic role of WARS2 in LUAD, we first evaluated its protein expression in clinical tissues. Immunohistochemical staining showed that WARS2 was weakly expressed in normal lung epithelium (**Figure 8A**), but was markedly upregulated in LUAD specimens (**Figure 8B**). Consistent with this observation, both qPCR and Western blot analyses confirmed that WARS2 expression was significantly reduced following siRNA-mediated knockdown in A549 and PC9 cells (**Figures 8C, D**).

Loss-of-function assays were then performed to assess the biological consequences of WARS2 silencing. EdU incorporation assays revealed that WARS2 knockdown markedly reduced the proportion of proliferating nuclei in both A549 and PC9 cells (**Figures 8E, F**). Similarly, colony formation assays demonstrated a dramatic decrease in clonogenic capacity upon WARS2 depletion (**Figures 8G, H**). In wound-healing and Transwell invasion assays, si-WARS2 cells exhibited substantially attenuated migratory and invasive abilities relative to the control (**Figures 8I, L**), and the corresponding quantitative analyses confirmed statistically significant reductions in migration and invasion rates (**Figures 8Q, V**).

To validate these findings *in vivo*, we established a xenograft model by subcutaneous injection of A549 and PC9 cells transfected with si-WARS2 or control siRNA into nude mice. Immunohistochemistry confirmed efficient WARS2 knockdown in tumor tissues (**Figures 8M, N**). Compared with controls, tumors derived from WARS2-silenced cells displayed markedly reduced volumes and weights (**Figures 8O–P**), supporting the growth-promoting function of WARS2 in LUAD.

Mechanistically, because WARS2 encodes a mitochondrial tryptophanyl-tRNA synthetase involved in oxidative phosphorylation (OXPHOS), we next examined whether its knockdown affects mitochondrial metabolic activity. Western blotting showed that silencing WARS2 led to a notable decrease in mitochondrial OXPHOS markers (COX I and ATP5A), accompanied by an increase in phosphorylated AMPK (p-AMPK) (**Figure 8W**), indicating impaired mitochondrial respiration and energy stress.

Finally, to determine whether WARS2 influences the tumor immune microenvironment, we analyzed immune cell populations in xenograft tumors by flow cytometry. The results revealed that WARS2 knockdown markedly increased CD3^+^CD8^+^ cytotoxic T-cell infiltration (**Figure 8X**) while decreasing CD45^+^CD206^+^ M2-like macrophages (**Figure 8Y**), as further quantified in **Figure 8Z**. These findings demonstrate that WARS2 contributes to the establishment of an immunosuppressive tumor microenvironment.

Collectively, these results confirm that WARS2 promotes LUAD progression by enhancing tumor cell proliferation and invasion, sustaining mitochondrial oxidative metabolism, and fostering an immune-suppressive microenvironment that favors tumor growth.

## 4 Discussion

In this study, we systematically constructed and validated a prognostic scoring model based on metabolic reprogramming features (MRPs), and further explored its biological underpinnings, immune landscape, intercellular communication patterns, and the functional role of the key driver gene WARS2 through multi-omics integration. Our overarching goal was to improve risk stratification and provide therapeutic guidance for lung adenocarcinoma (LUAD) patients. Although recent advances have been made in LUAD treatment and personalized medicine is gaining traction, current clinical practice still lacks a robust molecular-function-based classification system, contributing to substantial heterogeneity in patient outcomes (29). The MRPs scoring system proposed here aims to bridge this gap by incorporating transcriptional signatures of metabolic reprogramming, offering a novel prognostic dimension that complements traditional TNM staging and classical genetic biomarkers (e.g., EGFR, KRAS) (30).

Notably, the MRPs model demonstrated robust and independent prognostic value across the training, validation, and overall cohorts, with superior AUC values compared to TNM staging variables, highlighting its strong predictive accuracy. This finding suggests that transcriptomic features reflecting metabolic states may capture deeper biological traits beyond histological classification or driver mutations. For instance, the high-scoring MRP I group exhibited enhanced activity in pathways related to cell cycle, DNA replication, and protein synthesis, consistent with a more proliferative phenotype. Interestingly, such differences were not paralleled by tumor mutational burden (TMB) or major mutation profiles, implying that MRPs may reflect a non-genomic axis of malignant metabolic status.

Single-cell transcriptomic analysis further revealed that the metabolic states associated with MRPs are not limited to the bulk level but are also reflected in the cellular heterogeneity of LUAD. WARS2, identified as a key MRPG, was specifically enriched in malignant epithelial cells and correlated with elevated activities in oxidative phosphorylation, protein translation, and cell cycle processes. This aligns well with previous reports highlighting the role of aminoacyl-tRNA synthetase (ARS) family members in tumor metabolism and proliferation regulation (31–33). Functional comparisons between WARS2-positive and -negative cancer cells showed that the former exhibited higher metabolic activity and intercellular communication potential, particularly within adhesion-related pathways such as CD99, CDH, and FN1, potentially promoting cooperative tumor growth and immune evasion.

From an immunological perspective, the MRP I subgroup was characterized by an immunosuppressive phenotype, as evidenced by reduced ESTIMATE and ssGSEA immune scores and lower infiltration of key effector cells such as T cells and dendritic cells. The expression level of WARS2 was also consistent with this immunoevasive state, suggesting that WARS2 may be involved in reshaping the tumor immune microenvironment in LUAD. Previous studies have indicated that metabolic reprogramming can impair T-cell function and antigen presentation through mechanisms such as lactate accumulation, nutrient competition, and ROS modulation (34). Our findings suggest that WARS2 may represent a central node in these processes, warranting further mechanistic investigation.

Importantly, TIDE analysis suggested that MRP I patients may be more responsive to immune checkpoint inhibitors (ICIs), highlighting the potential influence of metabolic state on immunotherapy outcomes. WARS2 expression may thus serve not only as a metabolic marker but also as a predictor of immunotherapeutic efficacy. Furthermore, drug sensitivity analysis indicated that MRPs-based stratification holds promise for guiding personalized chemotherapy, though this requires further validation with pharmacological assays.

*In vitro* experiments confirmed the oncogenic role of WARS2 in LUAD. Silencing WARS2 significantly impaired cellular proliferation, colony formation, invasion, and migration in A549 and PC9 cells. *In vivo*, xenograft models demonstrated that WARS2 knockdown markedly suppressed tumor growth, providing functional evidence that WARS2 may act as a pro-tumorigenic factor and a potential therapeutic target.

Despite the consistent prognostic performance of the MRPs-based signature across multiple retrospective cohorts, our study has certain limitations. All current validations were conducted using public, retrospective datasets (TCGA and GEO), and the absence of a prospective, multi-center cohort limits the immediate generalizability of our findings. Recognizing this limitation, we have initiated the establishment of a prospective LUAD clinical cohort at our center, which will include transcriptomic profiling and detailed metabolic annotation. This ongoing effort aims to prospectively validate the MRPs signature and determine its utility in predicting patient outcomes and guiding therapeutic decision-making in clinical practice. Furthermore, integrating metabolomic and single-cell data from this cohort in future work may provide deeper mechanistic insights into metabolic reprogramming and immune regulation in LUAD.

In conclusion, our study is the first to systematically incorporate metabolic reprogramming features into the prognostic stratification of LUAD, and validate their biological and translational relevance through transcriptomic, single-cell, immunological, and functional analyses. This work establishes an integrative “metabolism–immunity–translation” framework for LUAD molecular research. Despite certain limitations—such as reliance on retrospective TCGA and GEO datasets, lack of prospective clinical validation, and the need for further mechanistic dissection of WARS2 function—our findings provide a compelling rationale for metabolic-state–based patient stratification and for the development of WARS2-targeted metabolic interventions in LUAD. Future efforts should focus on validating MRPs in clinical cohorts and exploring WARS2-directed therapies as a novel avenue for precision medicine in lung cancer.

## 5 Conclusions

In summary, this study proposed and validated a novel metabolic reprogramming-based prognostic scoring system (MRPs) for lung adenocarcinoma (LUAD), which demonstrated robust predictive accuracy and independent prognostic value beyond conventional clinical parameters. By integrating bulk and single-cell transcriptomic analyses, we revealed that high MRPs scores correspond to distinct malignant metabolic phenotypes characterized by enhanced proliferative signaling, immune evasion, and intercellular communication. We identified WARS2 as a key metabolic driver gene that is specifically enriched in cancer cells and functionally contributes to tumor growth and progression, as validated by both *in vitro* and *in vivo* experiments. Furthermore, immune landscape analysis and drug response prediction suggested that MRPs stratification may provide actionable insights into immunotherapy and chemotherapy sensitivity. Collectively, our findings establish a functional and clinically relevant molecular stratification framework grounded in tumor metabolic states, offering new perspectives for personalized risk assessment and therapeutic targeting in LUAD.

## Data Availability

Publicly available datasets were analyzed in this study. This data can be found here: https://xenabrowser.net/datapages/.

## Supplementary material

Supplementary Figure 1 **(A)** Volcano plot showing the differentially expressed genes (DEGs) between MRP I and MRP II subgroups in the TCGA LUAD cohort. Genes with |log_2_FC| > 1 and adjusted p < 0.05 were considered significant, with upregulated and downregulated genes labeled in red and blue, respectively. **(B)** Volcano plot of differentially expressed metabolic reprogramming-related genes (MRPGs) between MRP I and MRP II subgroups. **(C, D)** KEGG **(C)** and GO **(D)** enrichment analysis of DEGs from panel **(A)**, indicating enrichment in immune regulation, xenobiotic metabolism, and retinol-related pathways.**(E,F)** KEGG **(E)** and GO **(F)** enrichment analysis of differentially expressed MRPGs from panel **(B)**, highlighting subtype-specific metabolic alterations including glutathione metabolism, lipid metabolism, and oxidoreductase activity. **(G)** Circular bar plot of KEGG pathway terms for upregulated MRPGs, with color intensity indicating −log10(q-value) and bar length reflecting gene count. **(H)** Circular GO enrichment plot illustrating significantly enriched GO terms, with color-coded rings denoting ontology category and statistical significance.

Supplementary Figure 2 Violin plots show the expression levels of 21 key MRPGs in LUAD patients stratified by MRPs-based risk scores. Compared with the low-risk group, several MRPGs such as ARG2, CA7, CPS1, GSTA3, MTMR7, NT5E, and WARS2 were significantly upregulated in the high-risk group, while others like INPP5A, UGT2B11, and RDH16 showed modest or no significant differences. Statistical significance was determined using the Wilcoxon test (*P < 0.05; **P < 0.01; **P < 0.001; ns = not significant). These results further support the transcriptomic heterogeneity of metabolic reprogramming in LUAD.

Supplementary Figure 3 **(A–D)** Kaplan–Meier curves for overall survival (left) and disease-free survival (right) stratified by the expression of four metabolism-related genes in LUAD patients from TCGA. Patients were divided into high- and low-expression groups based on the median expression of each gene. **(A)** PDE11A expression was not significantly associated with survival outcomes. **(B)** RDH16 high expression was associated with worse overall survival (p = 0.031). **(C)** UGT2B11 showed no significant prognostic value. **(D)** WARS2 high expression was significantly associated with poorer overall survival (p = 0.0072), with a trend toward shorter disease-free survival (p = 0.057). These results highlight WARS2 as a potential prognostic marker in LUAD.

Supplementary Figure 4 **(A–C)** Distribution of MRPs scores (top panels) and corresponding survival status (bottom panels) for patients in the entire TCGA cohort **(A)**, training set **(B)**, and testing set **(C)**. Vertical dashed lines indicate the optimal cutoff separating MRP-I and MRP-II subtypes. **(D,E)** Similar univariate **(D)** and multivariate **(E)** Cox regression analyses in the TCGA testing cohort confirm the independent prognostic significance of the MRPs score. **(F)** Time-dependent ROC curves for the testing cohort also show good performance of the MRPs score in predicting 1-, 3-, and 5-year survival (AUC = 0.584, 0.706, and 0.716, respectively). **(G)** ROC comparison in the testing cohort confirms the superior predictive power of the MRPs score relative to individual clinical parameters.

Supplementary Figure 5 **(A)** Heatmap of GSVA scores for KEGG pathways significantly different between MRP I and MRP II subgroups. Metabolic pathways such as glycolysis, pyruvate metabolism, nucleotide metabolism, and cell cycle were differentially enriched. **(B)** Heatmap of GSVA scores for GO biological process terms significantly different between the two MRPs subgroups. Processes related to cell adhesion, extracellular matrix organization, and nucleoside biosynthesis showed subtype-specific enrichment.

Supplementary Figure 6 **(A–D)** Violin plots comparing ESTIMATEScore, TumorPurity, StromalScore, and ImmuneScore between MRP I and MRP II subgroups. MRP II samples exhibited significantly lower ESTIMATEScore, StromalScore, and ImmuneScore, and higher tumor purity (all p < 0.001). **(E)** Heatmap showing the relative abundance of immune-related functional modules and immune cell subsets across MRPs subtypes, suggesting distinct immune landscape between MRP I and MRP II. **(F–I)** Correlation between MRP score and ESTIMATEScore **(F)**, TumorPurity **(G)**, StromalScore **(H)**, and ImmuneScore **(I)**, indicating that higher MRP scores are associated with a more immune-depleted tumor microenvironment. **(J–M)** Correlation between WARS2 expression and ESTIMATEScore **(J)**, TumorPurity **(K)**, StromalScore **(L)**, and ImmuneScore **(M)**, showing that high WARS2 expression is similarly linked to lower immune and stromal content and increased tumor purity.
