# Supplementary figures and images for: Metabolic reprogramming-driven stratification and therapeutic targeting in lung adenocarcinoma: implications for prognosis and personalized treatment
Source: Front Oncol. 2026 Jan 5;15:1696117. doi: 10.3389/fonc.2025.1696117 (PMC12812633; doi:10.3389/fonc.2025.1696117)

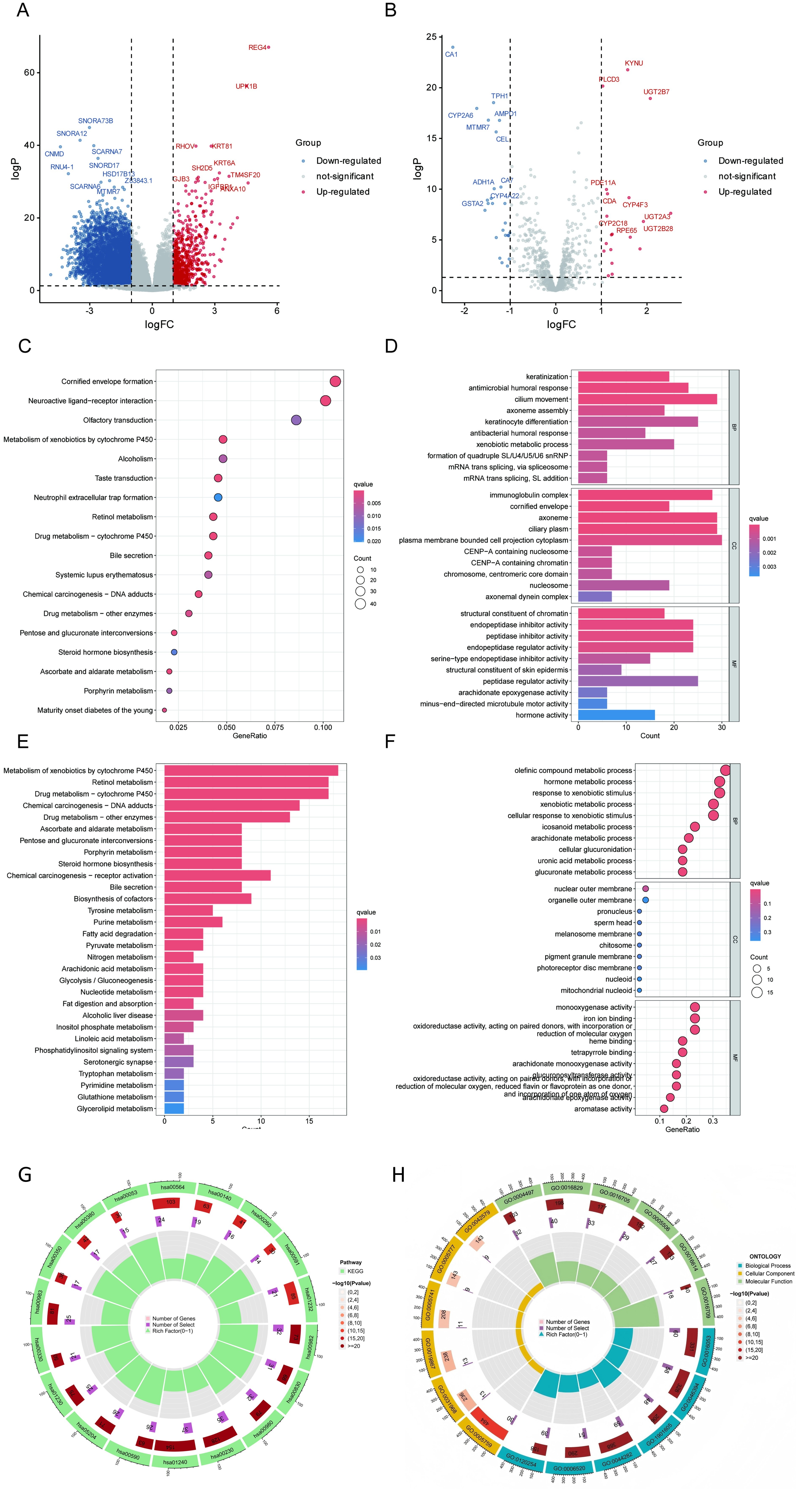

Supplement: Supplementary Figure 1 — (A) Volcano plot showing the differentially expressed genes (DEGs) between MRP I and MRP II subgroups in the TCGA LUAD cohort. Genes with |log2FC| > 1 and adjusted p < 0.05 were considered significant, with upregulated and downregulated genes labeled in red and blue, respectively. (B) Volcano plot of differentially expressed metabolic reprogramming-related genes (MRPGs) between MRP I and MRP II subgroups. (C, D) KEGG (C) and GO (D) enrichment analysis of DEGs from panel (A), indicating enrichment in immune regulation, xenobiotic metabolism, and retinol-related pathways.(E,F) KEGG (E) and GO (F) enrichment analysis of differentially expressed MRPGs from panel (B), highlighting subtype-specific metabolic alterations including glutathione metabolism, lipid metabolism, and oxidoreductase activity. (G) Circular bar plot of KEGG pathway terms for upregulated MRPGs, with color intensity indicating −log10(q-value) and bar length reflecting gene count. (H) Circular GO enrichment plot illustrating significantly enriched GO terms, with color-coded rings denoting ontology category and statistical significance. [file Image1.jpeg]

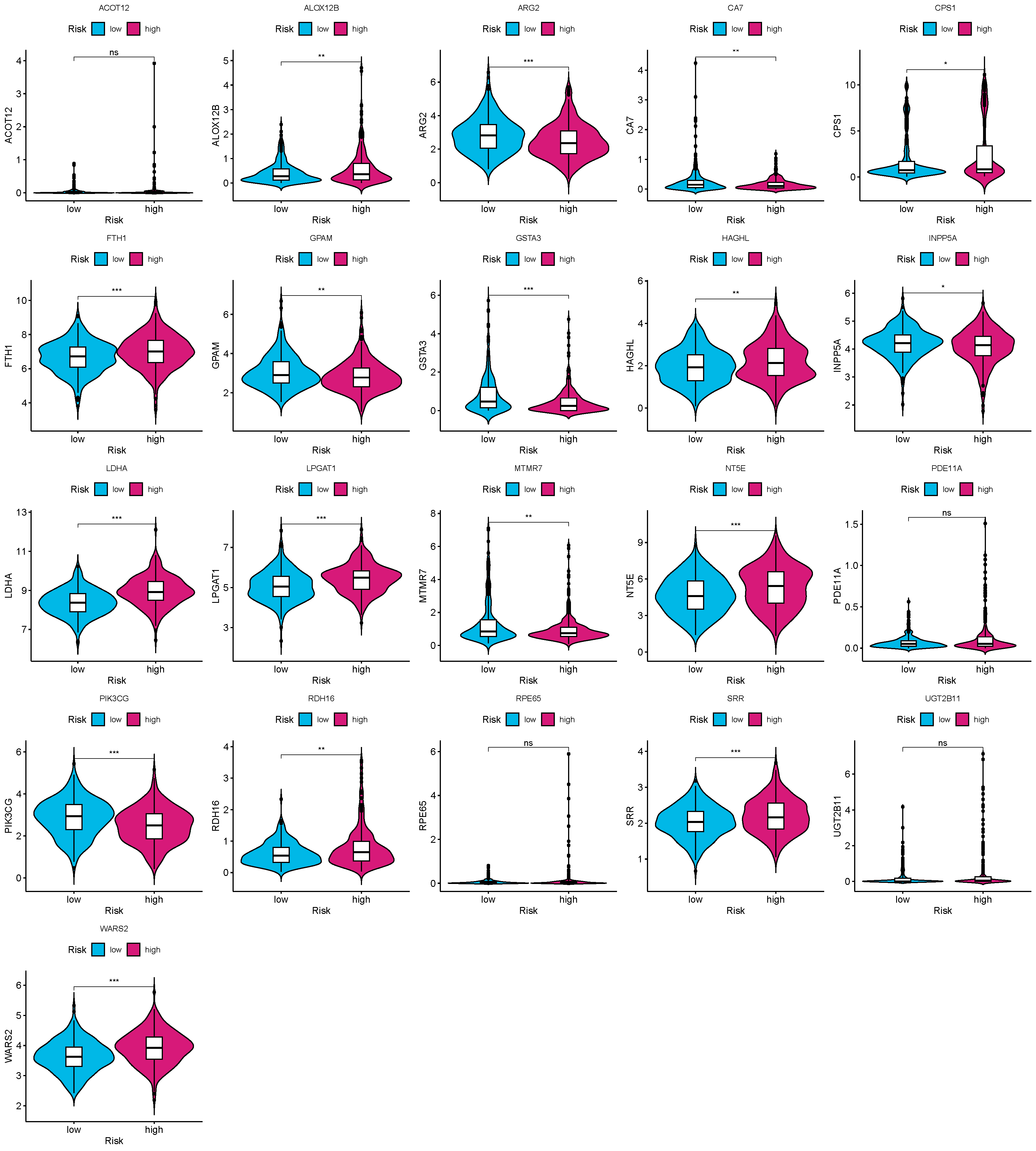

Supplement: Supplementary Figure 2 — Violin plots show the expression levels of 21 key MRPGs in LUAD patients stratified by MRPs-based risk scores. Compared with the low-risk group, several MRPGs such as ARG2, CA7, CPS1, GSTA3, MTMR7, NT5E, and WARS2 were significantly upregulated in the high-risk group, while others like INPP5A, UGT2B11, and RDH16 showed modest or no significant differences. Statistical significance was determined using the Wilcoxon test (*P < 0.05; **P < 0.01; **P < 0.001; ns = not significant). These results further support the transcriptomic heterogeneity of metabolic reprogramming in LUAD. [file Image2.jpeg]

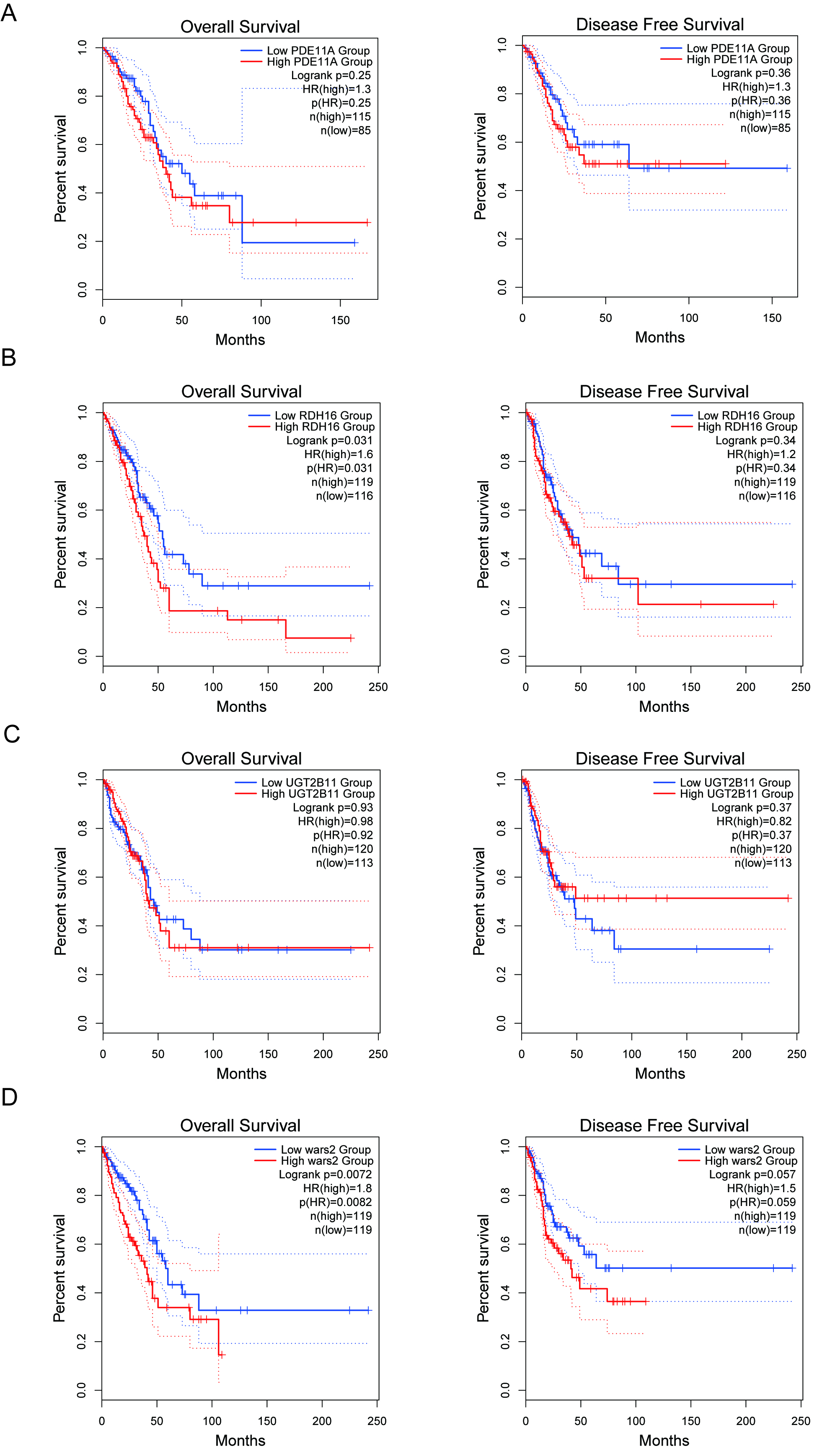

Supplement: Supplementary Figure 3 — (A–D) Kaplan–Meier curves for overall survival (left) and disease-free survival (right) stratified by the expression of four metabolism-related genes in LUAD patients from TCGA. Patients were divided into high- and low-expression groups based on the median expression of each gene. (A) PDE11A expression was not significantly associated with survival outcomes. (B) RDH16 high expression was associated with worse overall survival (p = 0.031). (C) UGT2B11 showed no significant prognostic value. (D) WARS2 high expression was significantly associated with poorer overall survival (p = 0.0072), with a trend toward shorter disease-free survival (p = 0.057). These results highlight WARS2 as a potential prognostic marker in LUAD. [file Image3.jpeg]

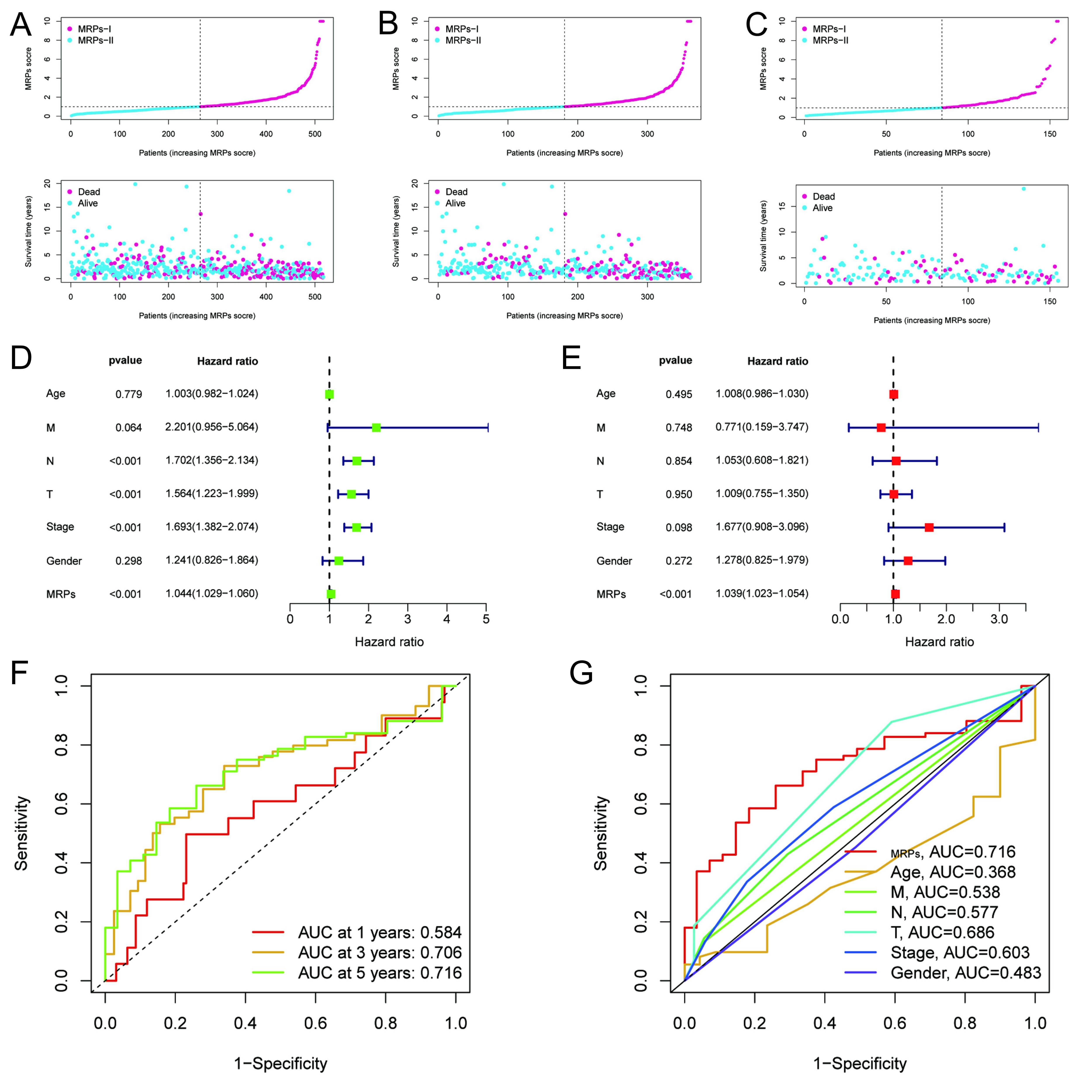

Supplement: Supplementary Figure 4 — (A–C) Distribution of MRPs scores (top panels) and corresponding survival status (bottom panels) for patients in the entire TCGA cohort (A), training set (B), and testing set (C). Vertical dashed lines indicate the optimal cutoff separating MRP-I and MRP-II subtypes. (D,E) Similar univariate (D) and multivariate (E) Cox regression analyses in the TCGA testing cohort confirm the independent prognostic significance of the MRPs score. (F) Time-dependent ROC curves for the testing cohort also show good performance of the MRPs score in predicting 1-, 3-, and 5-year survival (AUC = 0.584, 0.706, and 0.716, respectively). (G) ROC comparison in the testing cohort confirms the superior predictive power of the MRPs score relative to individual clinical parameters. [file Image4.jpeg]

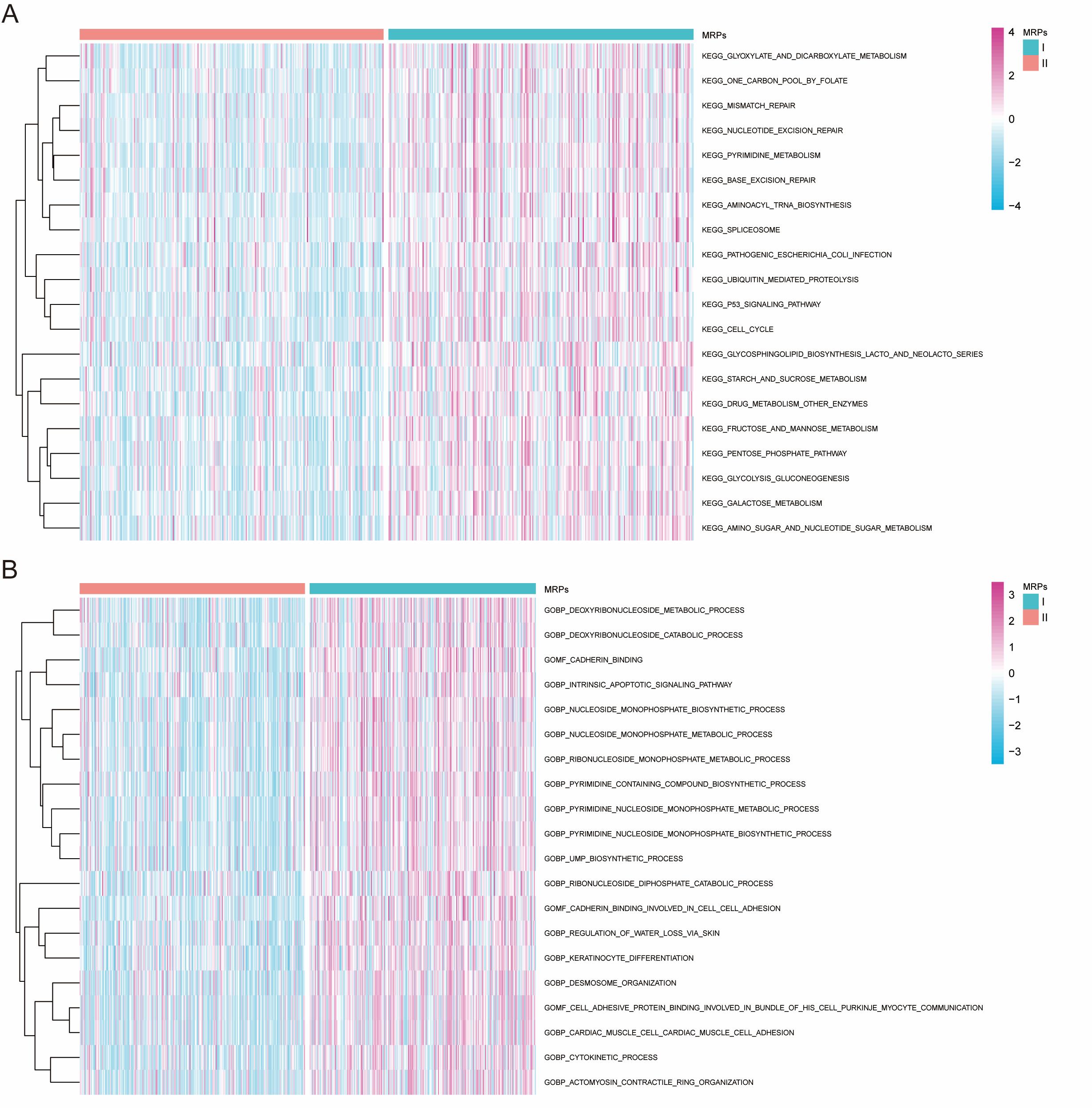

Supplement: Supplementary Figure 5 — (A) Heatmap of GSVA scores for KEGG pathways significantly different between MRP I and MRP II subgroups. Metabolic pathways such as glycolysis, pyruvate metabolism, nucleotide metabolism, and cell cycle were differentially enriched. (B) Heatmap of GSVA scores for GO biological process terms significantly different between the two MRPs subgroups. Processes related to cell adhesion, extracellular matrix organization, and nucleoside biosynthesis showed subtype-specific enrichment. [file Image5.jpeg]

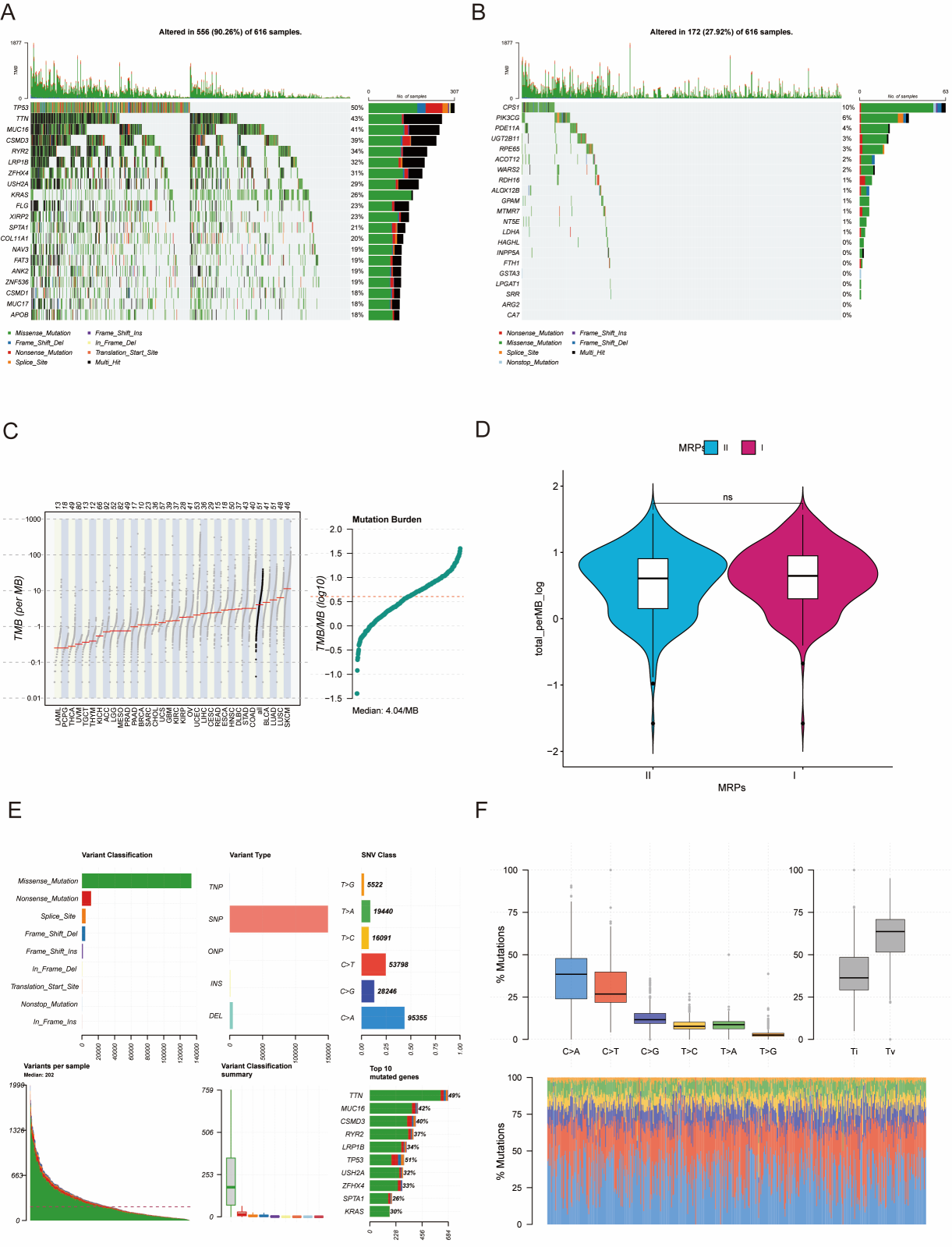

Supplement: Supplementary Figure 6 — (A–D) Violin plots comparing ESTIMATEScore, TumorPurity, StromalScore, and ImmuneScore between MRP I and MRP II subgroups. MRP II samples exhibited significantly lower ESTIMATEScore, StromalScore, and ImmuneScore, and higher tumor purity (all p < 0.001). (E) Heatmap showing the relative abundance of immune-related functional modules and immune cell subsets across MRPs subtypes, suggesting distinct immune landscape between MRP I and MRP II. (F–I) Correlation between MRP score and ESTIMATEScore (F), TumorPurity (G), StromalScore (H), and ImmuneScore (I), indicating that higher MRP scores are associated with a more immune-depleted tumor microenvironment. (J–M) Correlation between WARS2 expression and ESTIMATEScore (J), TumorPurity (K), StromalScore (L), and ImmuneScore (M), showing that high WARS2 expression is similarly linked to lower immune and stromal content and increased tumor purity. [file Image6.tiff]
